# Whole-genome de novo sequencing, combined with RNA-Seq analysis, reveals unique genome and physiological features of the amylolytic yeast *Saccharomycopsis fibuligera* and its interspecies hybrid

**DOI:** 10.1186/s13068-016-0653-4

**Published:** 2016-11-11

**Authors:** Jin Ho Choo, Chang Pyo Hong, Jae Yun Lim, Jeong-Ah Seo, Young-Suk Kim, Dong Wook Lee, Sin-Gi Park, Gir Won Lee, Emily Carroll, Yin-Won Lee, Hyun Ah Kang

**Affiliations:** 1 Department of Life Science, Chung-Ang University, Seoul, 06974 South Korea; 2 Theragen Bio Institute, TheragenEtex, Suwon, 16229 South Korea; 3 Department of Agricultural Biotechnology, Seoul National University, Seoul, 08826 South Korea; 4 School of Systems Biomedical Science, Soongsil University, Seoul, 06978 South Korea; 5 Department of Food Science and Engineering, Ewha Womans University, Seoul, 03760 South Korea

**Keywords:** Amylolytic yeasts, *Saccharomycopsis fibuligera*, Genome, Hybrid, RNA-Seq

## Abstract

**Background:**

Genomic studies on fungal species with hydrolytic activity have gained increased attention due to their great biotechnological potential for biomass-based biofuel production. The amylolytic yeast *Saccharomycopsis fibuligera* has served as a good source of enzymes and genes involved in saccharification. Despite its long history of use in food fermentation and bioethanol production, very little is known about the basic physiology and genomic features of *S. fibuligera*.

**Results:**

We performed whole-genome (WG) de novo sequencing and complete assembly of *S. fibuligera* KJJ81 and KPH12, two isolates from wheat-based *Nuruk* in Korea. Intriguingly, the KJJ81 genome (~38 Mb) was revealed as a hybrid between the KPH12 genome (~18 Mb) and another unidentified genome sharing 88.1% nucleotide identity with the KPH12 genome. The seven chromosome pairs of KJJ81 subgenomes exhibit highly conserved synteny, indicating a very recent hybridization event. The phylogeny inferred from WG comparisons showed an early divergence of *S. fibuligera* before the separation of the CTG and *Saccharomycetaceae* clades in the subphylum *Saccharomycotina*. Reconstructed carbon and sulfur metabolic pathways, coupled with RNA-Seq analysis, suggested a marginal Crabtree effect under high glucose and activation of sulfur metabolism toward methionine biosynthesis under sulfur limitation in this yeast. Notably, the lack of sulfate assimilation genes in the *S. fibuligera* genome reflects a unique phenotype for *Saccharomycopsis* clades as natural sulfur auxotrophs. Extended gene families, including novel genes involved in saccharification and proteolysis, were identified. Moreover, comparative genome analysis of *S. fibuligera* ATCC 36309, an isolate from chalky rye bread in Germany, revealed that an interchromosomal translocation occurred in the KPH12 genome before the generation of the KJJ81 hybrid genome.

**Conclusions:**

The completely sequenced *S. fibuligera* genome with high-quality annotation and RNA-Seq analysis establishes an important foundation for functional inference of *S. fibuligera* in the degradation of fermentation mash. The gene inventory facilitates the discovery of new genes applicable to the production of novel valuable enzymes and chemicals. Moreover, as the first gapless genome assembly in the genus *Saccharomycopsis* including members with desirable traits for bioconversion, the unique genomic features of *S. fibuligera* and its hybrid will provide in-depth insights into fungal genome dynamics as evolutionary adaptation.

**Electronic supplementary material:**

The online version of this article (doi:10.1186/s13068-016-0653-4) contains supplementary material, which is available to authorized users.

## Background

Genomic studies of the fungal species with hydrolytic activity have gained increased attention due to their great biotechnological potential in current and future biofuel production based on biomass [1]. *Saccharomycopsis fibuligera* (synonymous with *Endomyces fibuligera*), a member of the subphylum *Saccharomycotina* of the phylum *Ascomycota* of the *Fungi* kingdom, is found worldwide as the major amylolytic yeast utilized in indigenous food fermentation using rice and cassava. As a dimorphous yeast, *S. fibuligera* propagates by forming abundantly branched septate hyphae along with typical budding yeast-like cells [2]. This yeast has been considered one of the best producers of amylolytic enzymes among ascomycetous yeast species [3], since its capacity to perform starch hydrolysis was first reported by Wickerham et al. [4]. It is commonly found as a dominant yeast species in traditional Asian alcoholic starters for rice wine production, such as ‘*Daqu*’ in China [5], ‘*Ragi*’ in Indonesia [6], ‘*Loogpang*’ in Thailand [7], ‘*Banh Men*’ in Vietnam [8], and ‘*Nuruk*’ in Korea [9]. In addition, *S. fibuligera* was cultivated alone on starchy waste in Czechoslovakia or in mixed culture with *Candida utilis* on potato-processing wastes in Sweden to produce single-cell protein, which was used for protein supplementation in animal feeds [10]. Moreover, this yeast has been isolated as one of spoilage fungi causing chalk mold defects, which are commonly seen on the dark bread that is popular in continental Europe and the UK. It produces visible growth on the bread surface, exhibits a white and chalky appearance, and can spoil bread within a few days [11, 12].

*Saccharomycopsis fibuligera* has recently received increasing attention, as it produces trehalose, amylases, acid protease, and β-glucosidase, which have many applications in the food, fermentation, biofuel, and pharmaceutical industries [13–15]. The major role of *S. fibuligera* in the production process for traditional rice wine involves the conversion of starch into sugars, which can then be fermented into ethanol and organic acids [16]. It is noteworthy that some glucoamylases produced by *S. fibuligera* can digest native starch, which improves the degradation of starch from raw materials (i.e., barley and pea) in ‘*Daqu’* [13]. To exploit the strong amylolytic activity of *S. fibuligera* in utilizing starch as an inexpensive carbon source, synergistic co-cultures of *S. fibuligera* with a good ethanol producer, such as *Saccharomyces cerevisiae* or *Zymomonas mobilis,* have been grown to produce ethanol [17, 18]. Using cassava starch, *S. fibuligera* has been cultivated to produce amylolytic enzymes at industrial production levels [19]. Based on this high hydrolytic activity, *S. fibuligera* has recently been recognized as a potential industrial host for the bioremediation of agricultural waste [20]. *Saccharomycopsis fibuligera* has also served as a good donor of genes that are involved in saccharification to engineer *S. cerevisiae* for the development of a new yeast strain that can directly produce ethanol from biomass without the need for a separate saccharification process [21]. For example, the *S. fibuligera BGL1* gene, encoding β-glucosidase 1, has been heterologously expressed in *S. cerevisiae* to construct cellobiose-growing and fermenting strains [22–24]. Several studies aimed at overexpressing *S. fibuligera* β-glucosidase in heterologous hosts have been also conducted to provide the recombinant enzyme as supplement in cellulase mixtures to enhance the saccharification of cellulose [25, 26]. Although the production of lignocellulosic ethanol is expected to increase rapidly as advanced biofuel in the renewable fuel industry, starch is still the most commonly used feedstock for the production of conventional biofuel (http://www.afdc.energy.gov/laws/RFS). A recombinant yeast strain for the consolidated bioprocessing of starch biomass to ethanol, which can carry out raw starch hydrolysis and fermentation without any pretreatment of commercial enzyme addition, was recently developed by introducing the *S. fibuligera* α-amylase (*SFA1*) and *Thermomyces lanuginosus* glucoamylase (*TLG1*) genes into the industrial *S. cerevisiae* strains [27]. In addition to the bioconversion of starchy biomass to useful bioproducts, such as biofuels and trehalose, the high amylolytic activity of *S. fibuligera* can be usefully exploited in the production of other starch derivatives as well, including corn syrup, detergents, paper, textiles, and adhesive [28].

Despite the great potential of *S. fibuligera* as an industrial strain with a long history of use in various biotechnological applications and industrial fermentation, very little is known about the basic physiology and genomic features of *S. fibuligera*. In the present study, we conducted whole-genome (WG) de novo sequencing of two *S. fibuligera* isolates from *Nuruk,* a starter culture for the traditional rice wine *Makgeolli,* in Korea and performed a complete genome assembly with high-quality annotation, which was validated by RNA-Seq analysis. Furthermore, genome sequencing of another *S. fibuligera* isolate from chalky rye bread in Germany was performed and compared with the genomes of the *Nuruk* isolates. Our data revealed the unique genome features of *S. fibuligera* and its interspecies hybrid, which was generated by a very recent hybridization event. The completely sequenced and assembled *S. fibuligera* genomes with high-quality annotation, presented here, establish an important foundation for functional inference of this yeast and offer an interesting snapshot of the genomic evolutionary events that occurred after (inter)specific hybridization. Moreover, as the first gapless genome assembly reported in the genus *Saccharomycopsis* with desirable traits for bioconversion, it will serve as a reference genome to elucidate biological peculiarities specific to this yeast and its related lineage, facilitating the discovery of new genes applicable to the production of novel valuable enzymes and chemicals.

## Results

### Isolation and growth characteristics of *S. fibuligera* KJJ81 and KPH12 from *Nuruk*

**Fig. 1:**
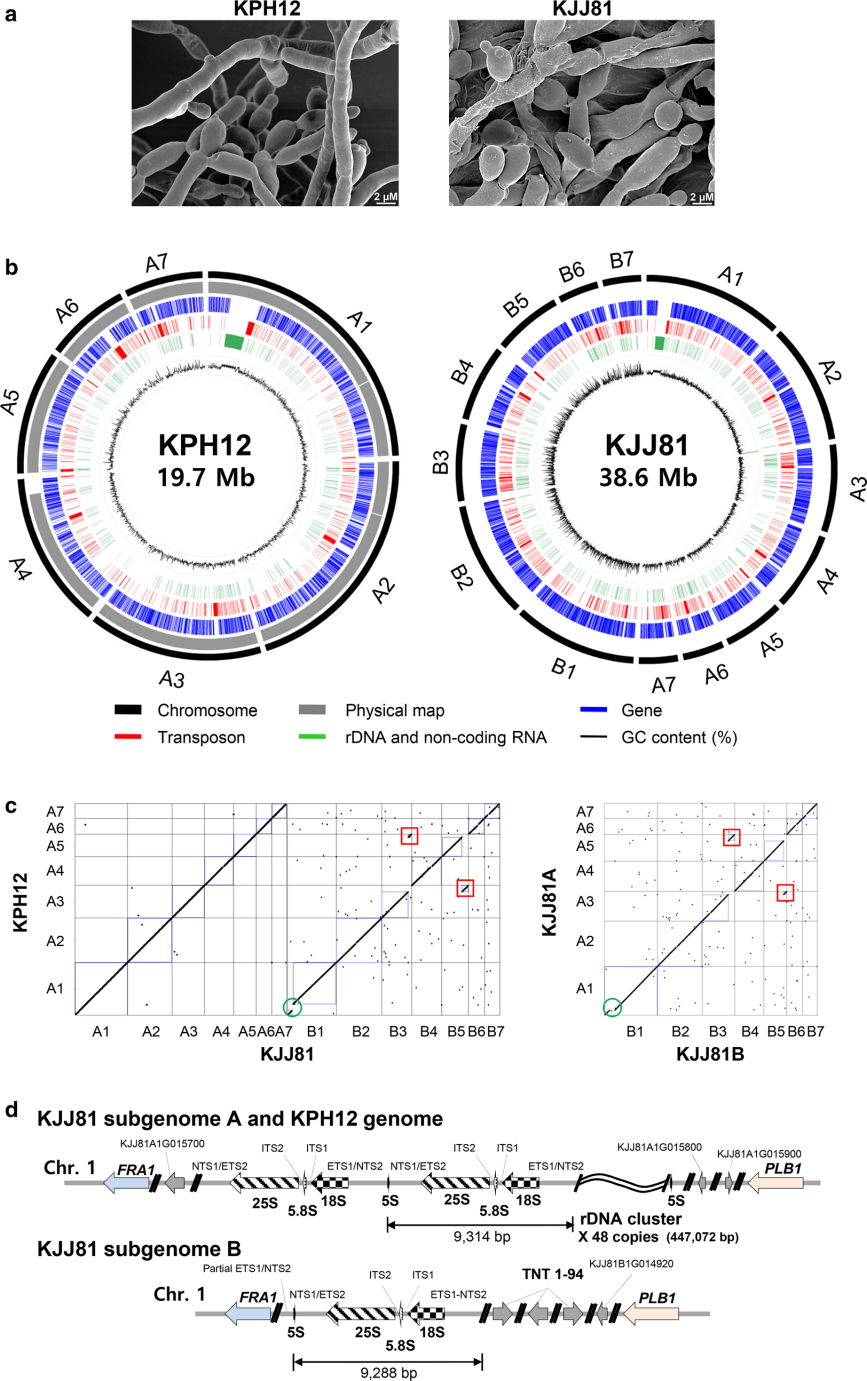
The morphology and genome organization of *S. fibuligera* KPH12 and KJJ81. **a** Filamentous growth of *S. fibuligera* KPH12 and KJJ81 with a minor fraction of budding yeast cells cultivated on YPD plates. **b** Diagram depicting the genome landscapes of KPH12 (*left*) and KJJ81 (*right*), with sequence coverage and similarity values. **c** Synteny analysis between the KPH12 and KJJ81 genomic sequences. WG dot plots of the two genomes were generated using SyMAP [82]. The *red boxes* indicate an interchromosomal translocation and the *green box* indicates a deletion. **d** Structure of rDNA repeats in the *S. fibuligera* genomes. ETS1, 5′-ETS (external transcribed spacer); ETS2, 3′-ETS; NTS, nontranscribed spacer; ITS, internal transcribed spacers; *FRA1*, an ORF encoding a putative Xaa-Pro aminopeptidase; TNT1-94, an ORF homologous to the retrovirus-related Pol polyprotein gene from *Nicotiana tabacum* transposon; *PLB1*, an ORF encoding lysophospholipase 1

Several fungal species were isolated from wheat-based *Nuruk* samples collected at various provinces in Korea. Among them, two yeast isolates, one from Jeju (KJJ81) and one from Pohang (KPH12), were chosen for further study based on their high saccharification activity. By analyzing the 5.8S ribosomal DNA (rDNA) sequences flanked by internal transcribed spacer (ITS) regions 1 and 2, both yeast isolates were identified as *S. fibuligera* and were thus named *S. fibuligera* KJJ81 and KPH12, respectively. The two yeast isolates *S. fibuligera* KJJ81 and KPH12 grow exclusively as filamentous forms with a minor fraction of budding yeast cells, and the hyphae of KJJ81 appear to be thicker than that of KPH12 (Fig. 1a).

The transition from yeast to hyphal growth was monitored by inoculation of the yeast-type cells of *S. fibuligera* KJJ81 and KPH12, which were enriched via filtration, into liquid YPD medium. With increased cultivation time, single yeast cells exhibited dimorphic growth, with a mixture of multipolar budding yeast cells, which are short ovals or long branched ovals, and septate mycelia. Nuclear Hoechst staining and calcofluor white staining for the septum and cell wall (Additional file 1: Figure S1, top) suggested that these hyphae-like structures were mostly pseudohyphae, which are actually chains of budded yeast cells that did not separate after duplication. Interestingly, we observed that yeast-like growth could be induced under carbon-limited culture conditions or in the presence of antimycin A, which blocks electron transport (Additional file 1: Figure S1, bottom). These observations suggest that the morphological shift (from yeast to hyphae and vice versa) of *S. fibuligera* is dependent on environmental conditions, such as nutrient- and energy-limited conditions, as has been observed for other dimorphic fungal species [29].

### Whole-genome de novo sequencing and assembly of *S. fibuligera* KJJ81 and KPH12

The WG de novo assemblies of *S. fibuligera* KJJ81 and KPH12 were produced from long-read single-molecule real-time and Illumina sequence data (Additional file 2: Tables S1–S4, Additional file 3: Figure S2). The final assembled KPH12 and KJJ81 genomes consisted of 7 and 14 linear scaffolds with N50 at 1.9 and 3 Mb, totaling 19.7 and 38.6 Mb, respectively (Table 1). The average sequence depth was 98.3× for KPH12 and 51× for KJJ81, respectively, with ≥99.97% genome coverage, indicating high accuracy of the assemblies (Additional file 4: Figure S3). In addition, TruSeq synthetic long-read (TSLR) assemblies for the two genomes supported the high concordance of the WG de novo assemblies with 96% coverage in the genome fraction. The accuracy of the scaffold order and the orientation of the KPH12 genome were further validated by optical mapping data [30]. The seven scaffolds of the KPH12 genome were anchored with 97.83% coverage to the 19 contigs assembled by BioNano optical mapping (Additional file 5: Figure S4). The optical mapping data showed the presence of one large deletion region of ~0.5 Mb in the largest scaffold assigned as chromosome 1 (chromosomes 1–7 in decreasing size from ~4.9 to ~1.3 Mb). The gap region on chromosome 1 was identified as the rDNA repeat locus with ~50 copies, estimated based on the size of the deletion region and the size of a single rDNA unit (9314 bp). The results strongly supported the notion that the seven scaffolds for the KPH12 genome were correctly assembled at the chromosome level (Fig. 1b, left).

**Table 1:** Summary of the *S. fibuligera* KPH12 and KJJ81 nuclear genome assembly and annotation

	KPH12	KJJ81
Scaffolds (no.)	7	14
Total length (bp)^a^	19,663,301	38,557,753
GC content (%)	38.24	38.54
Protein-coding gene models (no.)	6155	12,185
Unique gene models (no.)	6028	11,966
Genes with isoforms (no.)	127	219
Supported by RNA-Seq (no.)	6154	12,184
Annotated (no.)	5435	10,810
Average gene length (bp)	1788	1782
Total length of gene models (Mb)	11.00	21.72
Exons		
No. of exons	7710	15,067
No. of average exons per gene	1.25	1.23
Average exon length (bp)	1384	1402
Introns		
No. of introns	1555	2882
No. of average introns per gene	0.25	0.23
Average intron length (bp)	213	205
Non-coding RNAs (no.)		
tRNA	327	506
rRNAs	452	462
Average non-coding RNA length (bp)		
tRNA	83	84
rRNAs	464	463
Transposable element fragments (no.)		
Retrotransposons	1483	2691
DNA transposons	21	186
Mitochondrial genome		
Total length (bp)	67,427	67,516
GC content (%)	24.93	24.93

^a^Total length of scaffolds after extension of the telomere region

Quite intriguingly, comparative analysis of the KPH12 and KJJ81 genomic sequences revealed the co-linearity of two KJJ81 homologous scaffolds to each of the KPH12 pseudo-chromosomes (Fig. 1b, left). The 14 scaffolds of KJJ81 were classified into two subgenomes, designated A (an average similarity between KPH12 and A of 99.1%; totaling 19.7 Mb for seven scaffolds) and B (an average similarity between KPH12 and B of 88.6%; totaling 18.8 Mb for seven scaffolds) on the basis of sequence similarity between the two genomes. The similarity between two subgenomes of KJJ81was 89%. This result suggests that *S. fibuligera* KJJ81 is an adapted species containing seven chromosome pairs generated by hybridization between a progenitor of KPH12 and a divergent subspecies with approximately 88.6% nucleotide identity at the genome level.

### Annotation and synteny analysis of the *S. fibuligera* KJJ81 and KPH12 genomes

Employing ab initio and evidence-driven gene prediction methodology, we predicted a non-redundant set of 6155 protein-coding genes in the KPH12 genome, with an average length of 1.7 kb, an average exon length of 1.4 kb, and an average of 1.2 exons per gene. For the KJJ81 genome, 12,135 protein-coding genes were predicted with similar contents compared with those of KPH12 (Table 1). In the two genomes, the transcriptome (RNA-Seq) data generated in this study supported 98.7% of predicted gene models, and 89% of predicted genes had homology with gene models in the UniProt (77%), NCBI non-redundant (NR) (84%), and InterPro (85%) databases. Functional annotations were tentatively assigned to 64% of the genes (Additional file 2: Table S5).

Synteny analysis between the seven chromosomes of the KPH12 genome and KJJ81 subgenome A revealed that the chromosomes possess almost identical gene order. In contrast, a few noticeable alterations in genomic context, such as a reciprocal translocation between chromosomes 3 and 5 and a large deletion of the rDNA cluster encoding ribosomal RNA on chromosome 1, were observed in the synteny analysis between the KPH12 genome and KJJ81 subgenome B (Fig. 1c, left). Similarly, the seven chromosome pairs in subgenomes A and B of the KJJ81 hybrid genome completely preserved the conserved synteny, with the exception of the interchromosomal translocation between chromosomes 3 and 5 and the deletion of the rDNA cluster on chromosome 1 (Fig. 1c, right). Such a high level of synteny between the two subgenomes of KJJ81 strongly suggests that the hybrid event between the progenitor of KPH12 and its closely related subspecies took place very recently.

Complete sequences of the mitochondrial genomes (i.e., mtDNA) of KPH12 (67,427 bp) and KJJ81 (67,516 bp) were also determined (Table 1). Both *S. fibuligera* mtDNAs were almost identical at the nucleic acid sequence level, with a G-C content of 24.93%, except for one structural difference in the 5′ portion of one unidentified open reading frame (orf856 in KPH12 versus orf1057 in KJJ81) in the inverted repeat regions (Additional file 6: Figure S5a, b). The mtDNA contained at least 20 protein-coding genes (Additional file 2: Table S6), including the standard repertoire of yeast mtDNAs [31], along with 37 tRNA genes and 1 small subunit (*rrnS*)- and 2 large subunit (*rrnL*)-ribosomal RNA genes. *Saccharomycopsis fibuligera* mtDNAs contain genes encoding seven subunits of NADH dehydrogenase (nad1–6 and nad4L), which are not present in *Saccharomycetaceae* species, including the traditional yeast *S. cerevisiae* [32].

### Unique features of the *S. fibuligera* KJJ81 hybrid and KPH12 genomes

#### Ribosomal DNA clusters

The arrangement of the rRNA genes in *S. fibuligera* was revealed as similar to that in *S. cerevisiae* (Fig. 1d), in which a single unit consists of two transcribed regions, the 35S precursor rRNA- and 5S rRNA-coding regions, and two nontranscribed spacers (NTSs), NTS1 and NTS2. Both the KPH12 genome and KJJ81 subgenome A had rDNA loci consisting of ~50 copies of a repeating unit of 9314 bp on chromosome 1 (Fig. 1d, top panel). In contrast, KJJ81 subgenome B exhibited the deletion of the rDNA clusters, leaving only a single-copy rDNA unit of 9288 bp (Fig. 1d, lower panel). It is notable that there were three copies of retrotransposon elements, showing homology to tobacco TNT1–94, downstream of 18S rDNA in subgenome B of KJJ81 (Fig. 1d, lower panel). It can be speculated that the unilateral loss of rDNA clusters in subgenome B of KJJ81 might be mediated by transposon activity.

#### Putative centromere locations and telomere sequences

Members of the *Saccharomycetaceae* clade possess point centromeres with three characteristic conserved regions, whereas GC-poor troughs containing retrotransposon clusters have been suggested to mark the locations of centromeres in some yeast species [33]. Although the nucleotide GC composition in the *S. fibuligera* chromosomes was relatively uniform, without extended AT-rich regions (Additional file 7: Figure S6, black line), we observed a unique island of highly repeated and degenerate sequences of long terminal repeat (LTR) retrotransposons, belonging to the Ty1/*Copia* group, on each chromosome of KPH12 and KJJ81 (Additional file 7: Figure S6, red line). The clusters of direct and inverted repeats of LTR elements were identified at the same position on each chromosome pair, mostly in the middle of each *S. fibuligera* chromosome, with the exception of chromosome 5, suggesting that these retrotransposon clusters likely correspond to the centromere. The transcriptional profiles clearly showed a sharp and pronounced drop in the RNA-Seq signal strength with the minimum values at the predicted centromere regions in the plots (Additional file 7: Figure S6, green line). Thus, *S. fibuligera* centromeres are likely unique for each chromosome and marked by clusters of LTR sequences.

Sequence analysis of the extended end fragments of each *S. fibuligera* chromosome revealed the presence of “GGGTGGTGTAA” as the telomeric repeat sequences. It is noteworthy that the telomeric repeats of both *S. fibuligera* isolates contained ‘TGGTGT’, the most conserved motif observed among the various budding yeast species [34], but exhibited differences in the sequences flanking the conserved motif. We also observed the repeated sequences in slightly different patterns in the subtelomeric regions of *S. fibuligera* chromosomes (Additional file 8: Figure S7). The front, middle, and end blocks of the subtelomeric regions in subgenome B possessed repeat sequences that were distinct from those observed in subgenome A, reflecting subtelomeric polymorphisms between the two subgenome lineages. The presence of typical telomere and subtelomeric repeat sequences in each of the chromosomes strongly supports a complete and high-fidelity de novo genome assembly of *S. fibuligera* KPH12 and its hybrid isolate KJJ81.

#### Mating type locus and pheromone genes

The *S. fibuligera* KJJ81 and KPH12 genomes have two *MAT* loci, *MAT* **a** and *MAT*α, which exhibited very similar organization to those of methylotrophic yeast (Additional file 9: Figure S8a). The *MAT* **a** locus, containing *MAT* **a**1 and **a**2, was localized 170 kb away from the centromere of chromosome 4; the other *MAT*α locus, containing *MAT*α1 and α2, was present near the *MAT* **a** locus. The *SLA2*, which is a conserved gene found adjacent to the *MAT* loci in many yeasts and fungi [35], is also located beside the *MAT* **a** locus in *S. fibuligera.* However, the *S. fibuligera MAT* loci were not located in close proximity to the centromere or to the telomere and do not possess invertible sequences, which are observed in the methylotroph genomes [36], indicating that a different but as yet uncharacterized mechanism of switching and silencing is required for *S. fibuligera*.

Interestingly, the putative α-pheromone-encoding *MFα* genes of *S. fibuligera* were present on the other arm of chromosome 4, on which the *MAT* loci were clustered. It is quite noteworthy that the *MFα* genes in KJJ81 subgenomes A and B were quite distinctive, encoding two different precursor pheromone peptides with variations in spacer sequences and generating identical mature α-pheromone peptides, but with different copy numbers. The *MFα*1 gene (726-bp ORF) in KJJ81 subgenome B is expected to encode an α-factor precursor and will generate nine copies of the 11-amino acid mature peptide with the sequence “WAGVAPNQPIF” after proteolytic processing by the Kex2 protease and the Ste13 dipeptidyl aminopeptidase A. In contrast, the gene product of *MFα*2 (306-bp ORF) in KJJ81 subgenome A and the KPH12 genome is expected to generate only three copies of the mature *α*-peptide. The presence of different *MFα* genes in each of subgenomes A and B was clearly validated by PCR analysis using common forward and reverse primers designed based on the ORF sequences of MF*α*1 and *MFα*2 (Additional file 9: Figure S8b).

By searching for small encoded peptides with a terminal CAAX motif and an isoprenylation site, features that are conserved in the mature bioactive **a**-factors of several yeast species [37], we identified two putative *S. fibuligera MFA1 and MFA2* genes encoding two types of mature **a**-factors with one amino acid substitution at their C-termini, which were tandem clustered on chromosome 3 in KJJ8 subgenome A and the KPH12 genome. In contrast, KJJ81 subgenome B possessed only the *MFA1* gene with duplicate copies, thus producing only one type of **a**-factor (Additional file 9: Figure S8c). The **a**-factor of subgenome B demonstrated differences in two amino acid residues compared with those of subgenome A and the KPH12 genome. Such differences observed in the amino acid sequences and copy numbers of mating type pheromones between KJJ81 and KPH12 might affect mating efficiency and the cell cycle, which need to be investigated by performing more systematic experiments.

### Phylogenetic position of *S. fibuligera* based on genome comparison

**Fig. 2:**
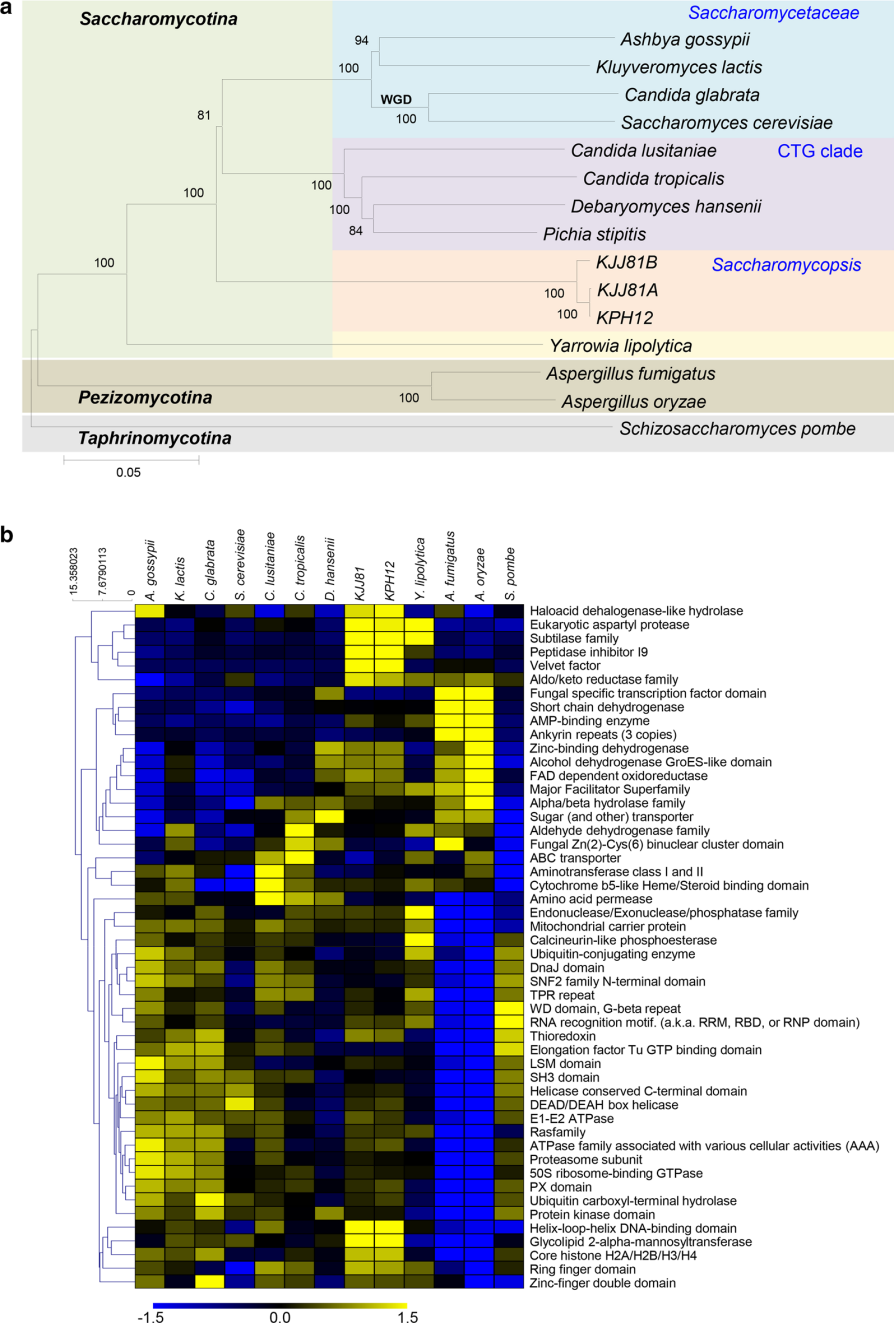
Phylogenetic and evolutionary analysis of *S. fibuligera* genomes in the phylum *Ascomycota*. **a** Phylogenetic tree analysis of KPH12 and KJJ81 based on genome sequence. A phylogenetic tree was constructed using MEGA6 [39] with other members of the phylum *Ascomycota,* which is composed of three major subphyla, *Pezizzomycotina, Saccharomycotina*, and *Taphrinomycotina.* A total of 55 orthologous genes were selected from among 12 ascomycete fungal species, including *A. gossypii*, *K. lactis*, *C. glabrata*, *S. cerevisiae*, *C. lusitaniae*, *C. tropicalis*, *D. hansenii*, *Y. lipolytica*, *A. fumigatus*, *A. oryzae*, and *S. pombe*. *WGD* whole-genome duplication event that occurred in a *Saccharomyces* species ancestor. **b** Expansion and contraction of protein families among the ascomycete fungal species, analyzed using the Pfam protein families database [84]

With the expectation that WG comparisons will provide far greater phylogenetic signals than the present five-gene dataset [38], we performed a phylogenetic analysis of *S. fibuligera* based on WG comparisons (Fig. 2a). Reciprocal pairwise comparison of the KPH12 and KJJ81 gene models with 12 WG sequenced fungal species of the phylum *Ascomycota* resulted in the identification of 55 orthologous genes that are commonly conserved in all of the analyzed species (Additional file 10). A phylogeny inferred on the basis of these orthology data positioned *S. fibuligera* as an early divergent of the subphylum *Saccharomycotina*, which was separated from a common ancestor much earlier before the divergence of *Saccharomycetaceae* and the CTG clades (Fig. 2a). Additionally, *S. fibuligera* was estimated to have diverged from *S. cerevisiae* approximately 164.5–194.1 Myr ago by calibrating the divergence times among *S. cerevisiae*, *Aspergillus oryzae*, and *Schizosaccharomyces pombe* using Molecular Evolutionary Genetics Analysis software MEGA6 [39]. The *S. fibuligera* genome rarely shares detectable syntenic gene blocks with any of these ascomycete fungal species, indicating its unique genome organization that was adapted after early divergence from a common ancestor of *Saccharomycotina*.

### Comparative gene inventories of *S. fibuligera*

The shared and unique protein families among fungal genomes are expected to be related to the histories and lifestyles of the compared fungal species. Therefore, we analyzed the contracted and extended Pfam domains in *S. fibuligera* relative to those of fungal species belonging to the phylum *Ascomycota* (Fig. 2b). Overall, the abundance of Pfam domains represented in *S. fibuligera* appeared to be quite unique in that several domains, including haloacid dehalogenase-like hydrolase, eukaryotic aspartyl protease, the subtilase family, peptidase inhibitor I9, velvet factor, helix-loop-helix DNA-binding domain, and glycolipid-2-α-mannosyltransferase, were particularly abundant in *S. fibuligera*. It is quite noteworthy that there was a significant overrepresentation of redundant gene families involved in protein degradation, such as eukaryotic aspartyl protease, the subtilase family, and peptidase inhibitor I9, in the *S. fibuligera* genomes. The expanded family of subtilins in *S. fibuligera* KPH12 consisted of 12 *PRB1* genes encoding vacuolar proteinase B, which is involved in the maturation of vacuolar aspartyl protease Pep4, four *KEX1* genes encoding a protease with a carboxypeptidase B-like function involved in killer toxin and α-factor precursor processing [40], and two *RRT12* genes encoding a protease with a role in spore wall assembly [41]. Most of these genes also contained the peptidase inhibitor I9 domain, which might be associated with the regulation of their own or other serine protease activities.

The velvet family of regulatory proteins plays a key role in coordinating development and secondary metabolism in response to light in several filamentous fungi carrying multiple light sensors that respond to different light wavelengths [42]. Such an extension of the velvet domain gene family, consisting of 12 genes encoding putative transcription factors, suggests that *S. fibuligera* might have evolved unique transcriptional regulation networks distinguishable from other yeast species. The significant expansions of the haloacid dehalogenase-like hydrolase (26 genes in KPH12) and the glycolipid 2-α-mannosyltransferase (12 genes in KPH12) gene families also suggest the peculiar physiological activity of *S. fibuligera,* which requires further study to be elucidated. Moreover, in part, the abundant patterns of some domains, including the aldo/keto reductase family, zinc-binding dehydrogenase, the alcohol dehydrogenase GroES-like domain, FAD-dependent oxidoreductase, and the major facilitator superfamily, were rather similar to those of *Aspergillus* species. This characteristic catalog for overrepresented and/or underrepresented protein domains would provide an important foundation for comparative biology and functional inference in *S. fibuligera,* elucidating biological peculiarities specific to *S. fibuligera* and its relative lineage.

### Reconstruction of the *S. fibuligera* C- and S-metabolic pathways with transcriptome profiles

#### C-metabolic pathway

**Fig. 3:**
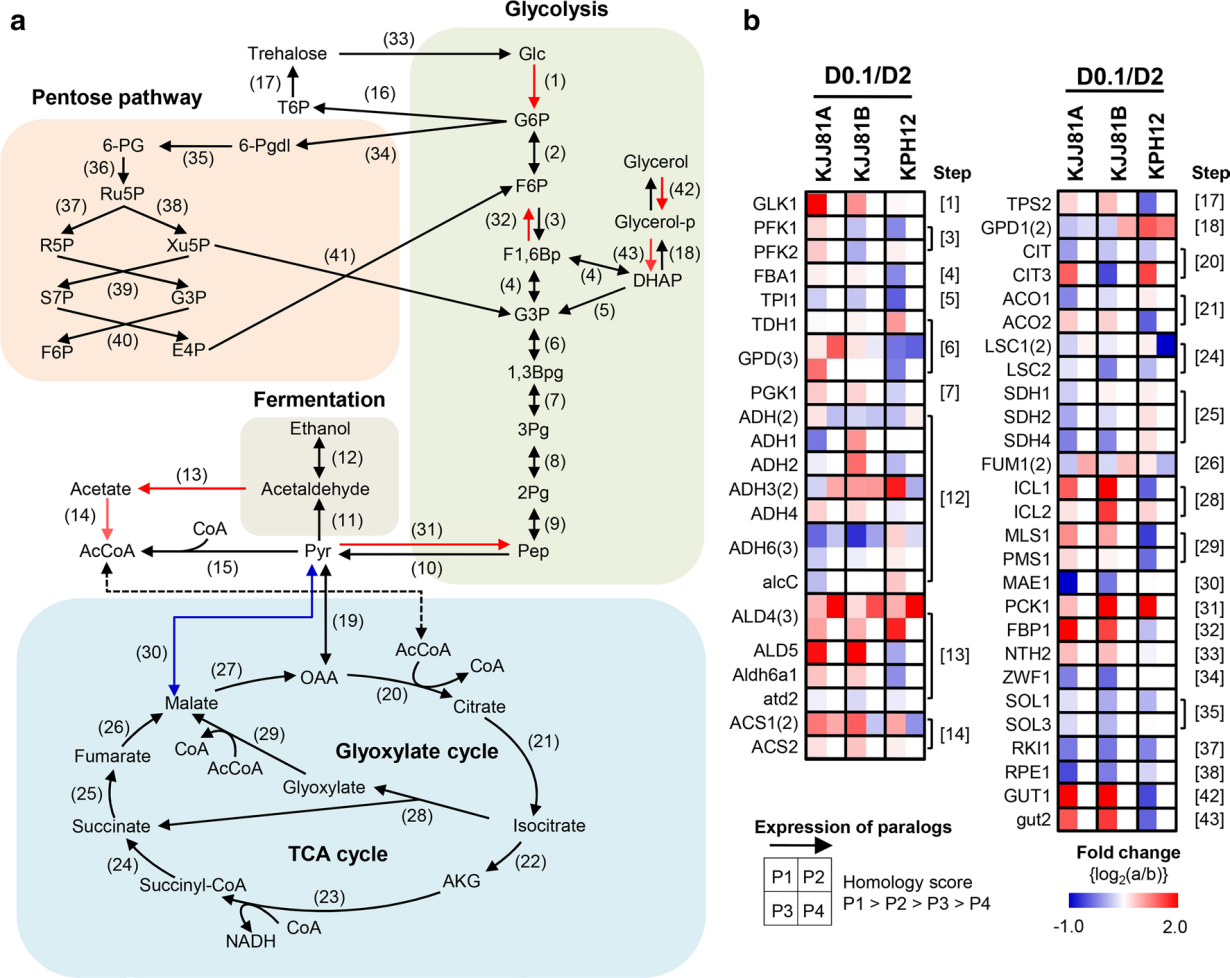
Reconstruction of the *S. fibuligera* carbon metabolic pathway mapped with transcriptome data. **a** *In silico* reconstructed carbon metabolic pathways of *S. fibuligera* based on annotated genome information. **b** Differential gene expression profiles of the C-metabolic pathway under low-glucose condition. D0.1, YPD containing 0.1% glucose; D2, YPD containing 2% glucose. The *number* in *parentheses* indicates the number of paralogs. Gene symbols, annotations, and expression abundance data are included in Additional file 11. The steps showing differential expression with a ≥1.5 fold change (the cutoff set at *P* < 0.05 in duplicated samples) under low-glucose condition (D0.1 versus D2) are indicated in *red* (induced) or *blue* (repressed) in the reconstructed C-metabolic pathway

Sugar metabolism provides an essential source of energy and metabolites for most organisms. The availability of the complete genome sequence of *S. fibuligera* allowed the identification of genes involved in glucose assimilation, including glycolysis, glyoxylate, the oxidative pentose phosphate pathway, the tricarboxylic acid cycle (TCA), and ethanol production (Fig. 3a). Transcript levels of genes involved in central carbon metabolism under high (2%, D2) and low glucose (0.1%, D0.1) were analyzed by RNA-Seq (Fig. 3b; Additional file 11). In contrast to *S. cerevisiae*, known as Crabtree-positive yeasts, which are subject to the glucose-mediated repression of respiration at high glucose levels [43], *S. fibuligera* did not show significant differences in the mRNA expression levels of the genes involved in the TCA cycle and cytochrome components under two culture conditions: 2% (D2) vs. 0.1% glucose (D0.1). In contrast, the genes encoding gluconeogenesis enzymes, including phosphoenolpyruvate carboxykinase (*PCK1*, step 31) and fructose-1,6-bisphosphatase (*FBP1*, step 32), showed apparently increased expression under 0.1% glucose compared with that under 2% glucose (Fig. 3b). These findings strongly indicate that *S. fibuligera* is highly likely to be a Crabtree-negative yeast, in which respiration capacity is not subject to glucose repression.

It is interesting to note that *S. fibuligera* possesses only the gene encoding glucose kinase (*GLK1*) and no genes encoding hexose kinases (*HXK1* and *HXK2*), which are required to initiate glucose assimilation and glucose phosphorylation (step 1). In contrast, *S. cerevisiae* possesses all three of these kinase genes, among which *HXK2* is reported to be mainly involved in glucose repression [43]. Moreover, *S. cerevisiae* possesses more than 20 *HXT* genes encoding hexose transporters, whereas *S. fibuligera* has only one homolog of *HXT5*, encoding a moderate-affinity glucose transporter, and four homologs for *HGT1*, encoding a high-affinity glucose transporter that is mostly found in Crabtree-negative yeasts and filamentous fungi [44]. In contrast, no low-affinity hexose transporter was predicted in the genome of *S. fibuligera*. It can be speculated that the lower numbers of genes involved in glucose transport and glucose phosphorylation for glycolysis might partly reflect the lesser degree of glucose repression in *S. fibuligera*. Along with the presence of the mitochondrial genes encoding NADH dehydrogenase in *S. fibuligera*, these observations explain that *S. fibuligera* is less efficient at ethanol fermentation than the good ethanol producer *S. cerevisiae*.

The marginal Crabtree effect was confirmed in *S. fibuligera* through the comparative analysis of glucose consumption and ethanol production under shake-flask cultivation with different glucose concentrations (Additional file 12: Figure S9). When cultivated in YP broth containing 0.1% glucose, ethanol production was not detected in either *S. cerevisiae* or *S*. *fibuligera* (Additional file 16: Figure S12a). However, in the presence of 2% glucose, ethanol accumulation with a maximum level of ~9 g/L was observed in *S. cerevisiae*, whereas ethanol was not detectable in *S. fibuligera* isolates KJJ81 and KPH12 (Additional file 16: Figure S12b). At much higher glucose concentrations, such as under cultivation in YP broth containing 10% glucose, *S. cerevisiae* was shown to efficiently convert glucose to ethanol, with up to ~46 g/L ethanol accumulating in the culture supernatant. In contrast, *S. fibuligera* KJJ81 and KPH12 accumulated ~15 and ~8 g/L ethanol, respectively, as the highest production levels (Additional file 16: Figure S12c). Compared with the Crabtree-positive yeast *S. cerevisiae*, *S. fibuligera* showed much less glucose consumption and ethanol production, while supporting cell growth comparably. This trend became stronger at high glucose concentrations. Interestingly, the *S. fibuligera* isolates KJJ81 and KPH12 showed different capacities of glucose consumption and ethanol fermentation, reflecting the differential gene expression profiles observed through RNA-Seq analysis (Fig. 3b).

#### S-metabolic pathway

**Fig. 4:**
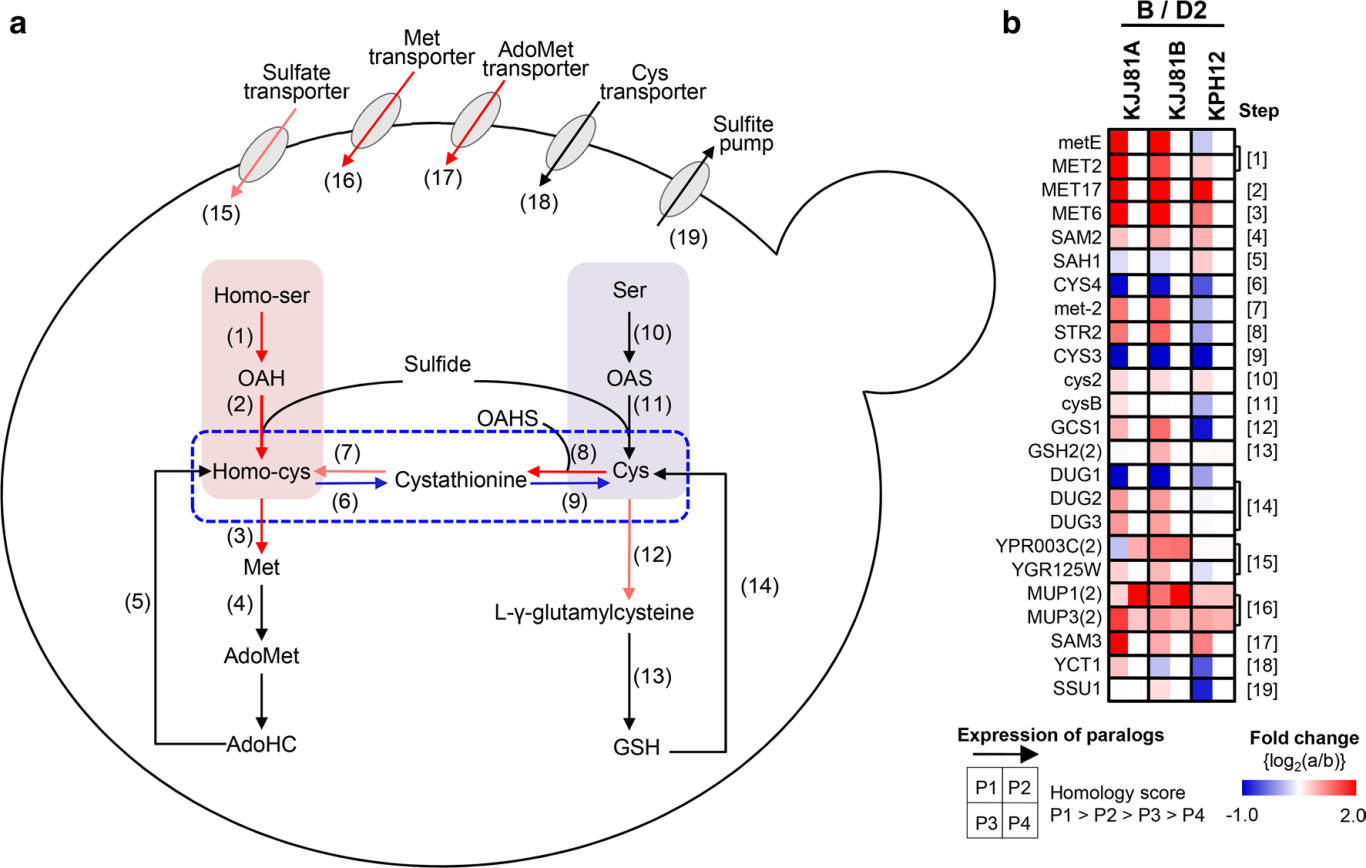
Reconstruction of the *S. fibuligera* sulfur assimilation pathway mapped with transcriptome data. **a** *In silico* reconstructed sulfur metabolic pathways of *S. fibuligera* based on annotated genome information. **b** Differential gene expression profiles of sulfur metabolic pathways under sulfur-limited condition. B, B medium containing 2% glucose; D2, YPD containing 2% glucose. The *number* in *parentheses* indicates the number of paralogs. Gene symbols, annotations, and expression abundance data are included in Additional file 11. The steps showing differential expression with a ≥1.5-fold change (the cutoff set at *P* < 0.05 in duplicated samples) under sulfur-limited condition (B versus D2) are indicated in *red* (induced) or *blue* (repressed) in the reconstructed S-metabolic pathway

Cellular requirements for sulfur can be fulfilled by the uptake of sulfur-containing amino acids, or by the assimilation of inorganic sulfur into organic compounds [45]. It is notable that all known species of the *Saccharomycopsis* clade are reported to be deficient in sulfate uptake and require supplementation with one of a variety of organic sulfur sources [46]. Intriguingly, our genome data revealed the presence of ORFs encoding uncharacterized sulfate transporters and a sulfite pump, but the absence of ORFs showing homology to other essential genes for sulfate assimilation, such as *MET3* (sulfate adenylyltransferase), *MET14* (adenylyl-sulfate kinase), *MET16* (phosphoadenylyl-sulfate reductase), and *MET5*/*ME10* (sulfite reductase alpha/beta), in the genomes of *S. fibuligera* KJJ81 and KPH12 (Fig. 4a).

In yeast and fungal species, cysteine biosynthesis from sulfide can be divided into two pathways. In one pathway, sulfide is condensed with *O*-acetylserine to generate cysteine in a process catalyzed by cysteine synthase (OAS pathway). In the other pathway, sulfide is condensed with *O*-acetylhomoserine to generate homocysteine (OAH pathway), which can be converted to cystathionine and then to cysteine via a transsulfuration pathway [47, 48]. In contrast to *S. cerevisiae*, which lacks the OAS pathway, but similar to filamentous fungal species employing both pathways for cysteine biosynthesis, *S. fibuligera* has genes encoding a direct pathway to cysteine in addition to a transsulfuration pathway from homocysteine. The transcriptome profile analysis revealed that the sulfur pathway toward methionine biosynthesis, such as those genes encoding methionine transporters (steps 16 and 17) and the interconversion of cysteine to methionine pathways (steps 7 and 8) and the OAH pathway (steps 1, 2, and 3), was obviously activated when *S. fibuligera* was cultivated on sulfur-limited B medium compared with cultivation on rich YPD medium (Fig. 4b; Additional file 11). In contrast, the transsulfuration pathway for the biosynthesis of cysteine from homocysteine (steps 6 and 9) is dramatically depressed under sulfur-limited conditions. This finding indicates the presence of a regulatory system in *S. fibuligera* to activate and turn off the sulfur assimilation pathway toward the biosynthesis of methionine, depending on the presence of readily usable sulfur compounds (Additional file 11).

### Extracellular enzymes of *S. fibuligera* with biotechnological applications

#### Cellulose degradation enzymes

**Fig. 5:**
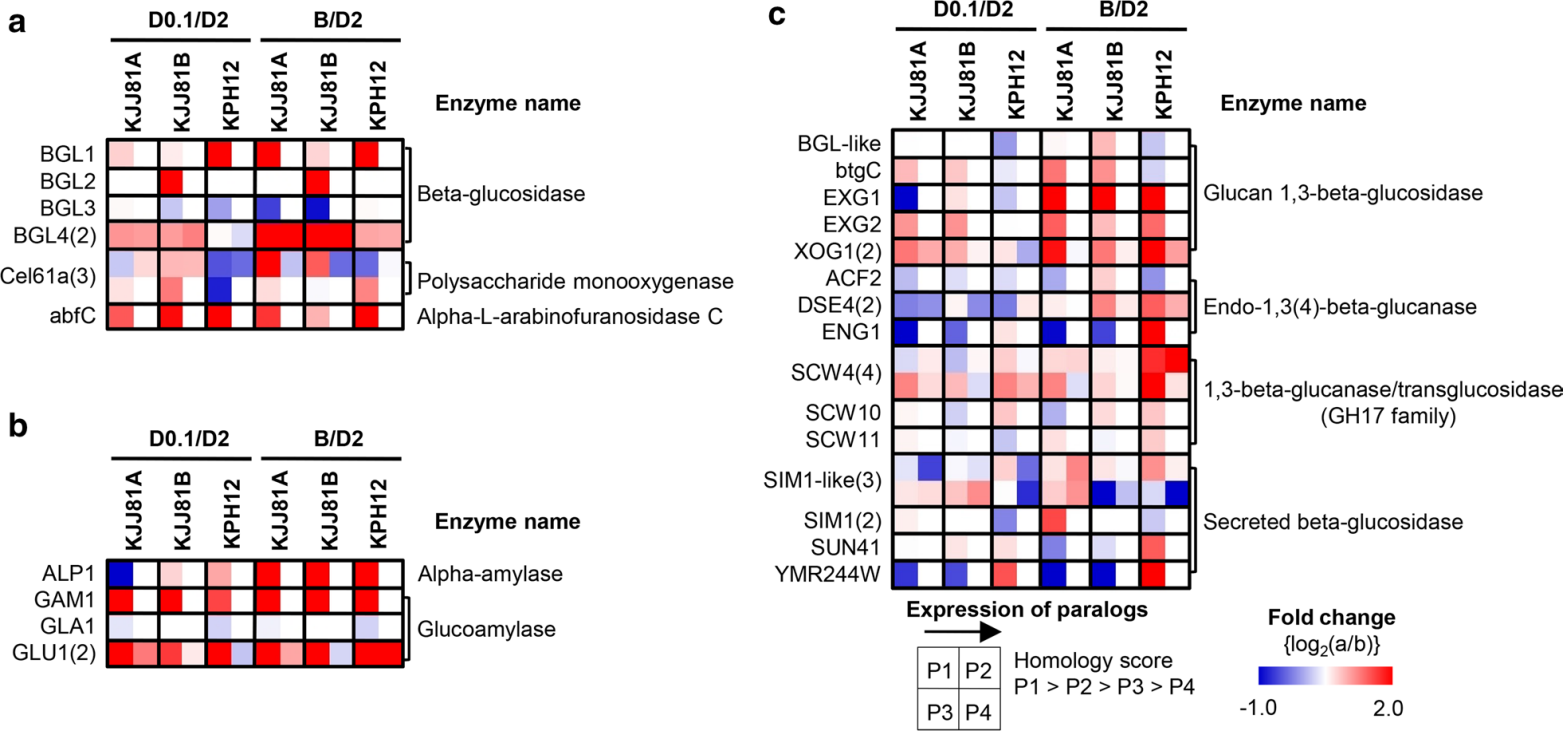
Expression profiles of *S. fibuligera* genes associated with cellulose, starch, and cell wall degradation. Differential gene expression profiles of genes in the KJJ81 and PH12 genomes that are associated with the degradation of cellulose (**a**), starch (**b**), and the cell wall (**c**). D2, YP containing 2% glucose; D0.1, YP containing 0.1% glucose; B, B medium containing 2% glucose. The *number* in *parentheses* indicates the number of paralogs. Gene symbols, annotations, and expression abundance data are included in Additional file 13

The enzymatic degradation of cellulose, the most abundant polymer on earth, includes the joint action of exoglucanases or cellobiohydrolases, endoglucanases, and β-glucosidases (BGL). The hydrolytic enzymes have attracted intensive research interest due to their use in lignocellulosic biomass decomposition for the production of biofuels and high-value chemicals. Analysis of the *S. fibuligera* genome revealed that this yeast retains several genes encoding hydrolytic enzymes, which are found in the majority of cellulolytic fungi (Fig. 5a). There were four or five copies of the genes encoding BGL, which acts mainly on cellobiose, in the *S. fibuligera* genome. Comparison of the *S. fibuligera* KJJ81 and KPH12 genomes unveiled quite interesting differences in the composition of the BGL family: the gene encoding β-glucosidase 1 (*sfBGL1*, P22506.1) is present in all three genomes, whereas the gene encoding sfBGL2 (*sfBGL2*, P22507.1) is absent in subgenome A of *S*. *fibuligera* KJJ81 and the KPH12 genome. A novel gene encoding a protein with considerable homology to SfBGL1 (55% identity), designated *sfBGL3*, and two additional genes encoding homologs of *S. pombe* putative β-glucosidase, designated *sfBGL4,* were discovered in all genomes of *S. fibuligera.* The catalytic activities of those novel β-glucosidases must be investigated and compared with BGL1 and BGL2.

Furthermore, three *S. fibuligera* homologs to the thermophilic fungus *T. reesei* and *Sporotrichum thermophile* Cel61A proteins, formerly known as GH61 proteins with β1,4 endoglucanase activity, were annotated. Recent studies have reported that *T. reesei* and *S. thermophile* Cel61A are actually copper-dependent polysaccharide monooxygenases (PMOs), which constitute a novel class of enzymes that catalyze the O_2_-dependent oxidative cleavage of recalcitrant polysaccharides [49]. PMOs are of increased biotechnological interest because they boost the efficiency of common cellulases, resulting in increased hydrolysis yields while reducing the protein loading needed for plant polysaccharide degradation. Another interesting observation is the presence of a homolog of *A. nidulans* abfC, encoding a probable α-l-arabinofuranosidase C involved in the degradation of hemicellulose polymers with arabinosyl resides, in the *S. fibuligera* KJJ81 and KPH12 genomes. The expression of this *S. fibuligera* abfC homolog is highly induced under glucose-limited and sulfur-limited conditions (Additional file 13). Retention of a subset of cellulose degradation genes in *S. fibuligera* is quite noteworthy, in that most yeast species belonging to the *Saccharomycotina* subphylum have lost the gene sets involved in cellulose degradation. Biochemical characterization is required to define their substrate specificity and the biological functions of putative *S. fibuligera* Cel61A and abfC proteins.

#### Amylolytic enzymes for starch degradation

Starch is a practical substrate for the production of yeast cells and their fermentation products on a large scale due to its low price and the easy availability of raw material in most regions of the world. *S. fibuligera* can degrade starch with great efficacy because it expresses glucoamylase, catalyzing the release of glucose from the non-reducing ends of starch molecules, in addition to α-amylase, catalyzing the cleavage of 1,4-α-glycosidic bonds in starch. Only one gene encoding a secretable α-amylase (*ALP1*), whose deduced sequence was previously reported to be highly homologous with that of α-amylase from *A. oryzae* [50], was found in the *S. fibuligera* KJJ81 and KPH12 genomes (Fig. 5b). The *GLA1* and *GLU1* genes, encoding two types of glucoamylases, Gla and Glu, reported in *S. fibuligera* KZ and HUT7212, respectively [51], and an ORF, homologous to the *C. albicans* glucoamylase gene (*GAM1*), were identified in our *S. fibuligera* genomes. However, the *GLA1* gene appeared to be lost in *S. fibuligera* KJJ81 subgenome B (Additional file 13). Moreover, the *GLM1* gene (EMBL accession no. AJ311587) encoding the raw starch digestion glucoamylase Glm in *S. fibuligera* IFO 0111 [52] was not identified in the genomes of *S. fibuligera* KJJ81 and KPH12, indicating that *S. fibuligera* IFO 0111 might be unique in the repertories of the amylolytic enzyme complex. Interestingly, the expression of these amylolytic enzyme genes, except for *GLA1*, was observed to be highly induced under low-glucose and sulfur-limitation conditions.

#### Cell wall degradation enzymes

Another notable observation in the *S. fibuligera* genome was the presence of redundant genes related to the degradation of β-glucan, the major component of the fungal cell wall and grains. The major cell wall polysaccharide in the endosperm of cereals is a linear β-1,3(4)-d-glucan (β-glucan), which accounts for up to 5.5% of the dry weight of grains [53]. There were multiple genes, annotated as glucan 1,3-β-glucosidase, endo-1,3(4)-β-glucanase, 1,3-β-glucanase/transglucosidase, and secreted β-glucosidase, which are involved in cell wall maintenance and cytokinesis (Fig. 5c; Additional file 13). In particular, β-1,3(4)-β-glucanases (EC 3.2.1.73; lichenase), which strictly cleave the β-1,4-glycosidic linkage adjacent to a 3-*O*-substituted glucose residue in mixed linked β-glucans, are important biotechnological aids in the brewing and animal feed stuff industries [54].

#### Acidic proteases

**Fig. 6:**
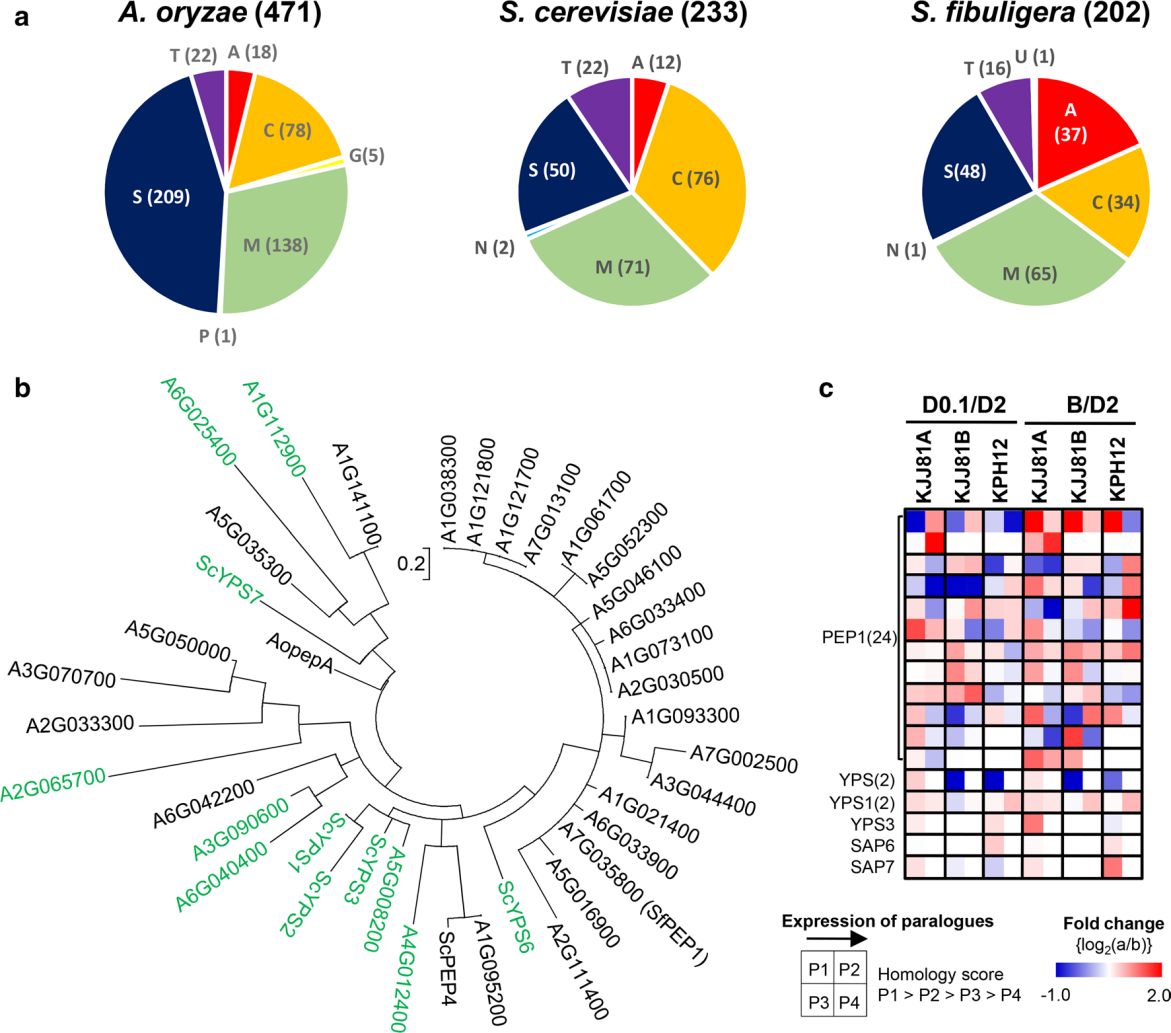
Analysis of putative protease genes in *S. fibuligera*. **a** Pie charts of the putative protease family of *A. oryzae*, *S cerevisiae*, and *S. fibuligera* KPH12. Families of proteolytic enzymes are grouped according to the peptidase database MEROPS (https://merops.sanger.ac.uk/). *A* aspartic, *C* cysteine, *G* glutamic, *M* metallo, *N* asparagine, *P* mixed, *S* serine, *T* threonine, *U* unknown proteases. The numbers of genes assigned to each of the families are shown. **b** Phylogenic tree of secretory proteases in KJJ81 subgenome A. *Saccharomyces cerevisiae* and putative *S. fibuligera* yapsins carrying the GPI motif are shown in *green*. *A. oryzae* acidic protease (*AopepA*, XP_001824175.1), *S. cerevisiae* vacuolar acidic protease *PEP4* (*ScPEP4*, CAA97859.1), *S. cerevisiae* yapsin proteases, *ScYPS1* (KZV09366.1), *ScYPS2* (KZV12382.1), *ScYPS3* (KZV09368.1), *ScYPS6* (KZV10664.1), and *ScYPS7* (KZV12588.1) are included for comparison. **c** Transcriptional analysis of the *S. fibuligera* genes encoding secreted acidic proteases. D2, YP containing 2% glucose; D0.1, YP containing 0.1% glucose; B, B medium containing 2% glucose. The *number* in *parentheses* indicates the number of paralogs. Gene symbols, annotations, and expression abundance data are included in Additional file 13

As indicated in Fig. 2, which shows the contracted and extended Pfam domains in the *S. fibuligera* genome, the aspartic protease family genes were observed to be particularly enriched in *S. fibuligera* compared with *A. oryzae* and *S. cerevisiae* (Fig. 6a). Notably, the genome of *S. fibuligera* KPH12 contained more than 37 ORFs encoding putative acid proteases, 31 of which encoded extracellular secretory acid proteases (Additional file 13). The predicted secretory acidic proteases carry a hydrophobic amino-terminal segment as a secretion signal sequence and contain a catalytic active domain surrounding the two active-site aspartate residues, which demonstrate significant homologies to the aspartyl protease family. The secretory acid proteases, with optimal pH values in the acidic range (pH 3–4), play important roles in hydrolyzing proteins in the fermentation mash to liberate amino acids or peptides under the acidic conditions of fermentation on mash [55].

The first identified *PEP1* gene, encoding a secretory acid protease in *S. fibuligera* A11 (synonymous to *APG*, accession number E01179), does not contain a glycosylphosphatidylinositol (GPI) anchor motif [56]. While 24 genes among a total of 31 *S. fibuligera* genes encoding secretory acidic protease were grouped as *PEP1* paralogs, 7 genes were analyzed and found to encode secretory proteases containing the GPI anchor motif at their C-termini, as reported in the *S. cerevisiae* yapsin and *Candida albicans* sap proteases, which are mostly localized at the cell surface [57]. Therefore, genes encoding GIP-anchored yeast aspartyl protease members are annotated as paralogs of yapsin-like proteases (Fig. 6b, indicated in green). Phylogenetic tree analysis suggested that the *PEP1* family members might have diverged from yapsin-like proteases via loss of the GPI anchor motif. It appeared that the expression of some *PEP1* family members was highly induced under low-glucose and sulfur-limited conditions, whereas the expression of yapsin-like proteases was mostly constitutive, without significant changes at the transcription level (Fig. 6c; Additional file 13).

### Comparative genome analysis of an *S. fibuligera* isolate from chalky rye bread in Germany

**Fig. 7:**
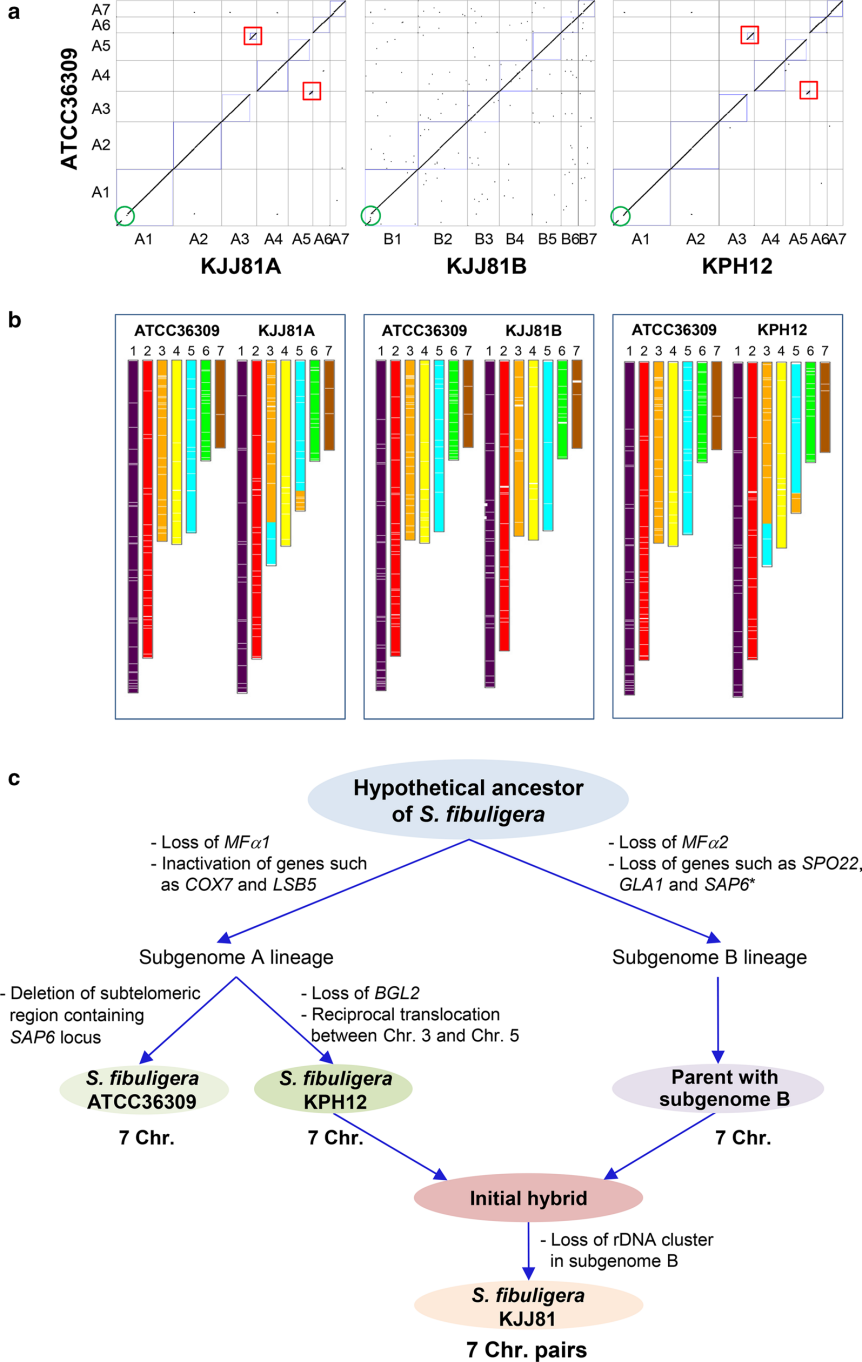
Comparative analysis of the *S. fibuligera* ATCC 36309, and KPH12 and KJJ81 genomes. **a** Synteny analysis between the two genomic sequences (*top*) with information on sequence coverage and similarity of the *S. fibuligera* genomes (*bottom*). WG dot plots of the two genomes were generated using SyMAP. The *red boxes* indicate an interchromosomal translocation and the *green box* indicates a deletion. **b** Synteny blocks between the two genomic sequences were visualized through chromosome painting in SynChro [85]. **c** Hybrid formation between two *S. fibuligera* subspecies with subgenomes A and B, respectively. Two subspecies were separated from the last common ancestor of *S. fibuligera* by various genetic changes. In the subgenome A lineage, *S. fibuligera* KPH12 diverged from *S. fibuligera* ATCC 36309 with additional genetic changes, such as the loss of *BGL2* and reciprocal translocation between chromosomes 3 and 5. Hybridization of *S. fibuligera* KPH12 and a parent with subgenome B, followed by loss of the rDNA cluster in subgenome B, might generate the *S. fibuligera* KJJ81 hybrid carrying seven chromosome pairs. Loss of some non-essential genes in the subgenome B lineage could occur after a hybridization event (*asterisk*)

Genome sequencing provides the most complete understanding of the genome structure of an organism and allows for the most in-depth comparisons to be made between related species and even between different strains of one species that have been isolated from varied environmental conditions. Therefore, we undertook WG de novo assembly of *S. fibuligera* ATCC 36309, an isolate from chalky rye bread in Germany, from long-read SMRT and Illumina sequence data. A total of seven scaffolds were assembled on the basis of KPH12 assembly data, estimated to be approximately 19.6 Mb in length (Additional file 2: Table S7). Ab initio gene prediction using extrinsic evidence extracted from KPH12 and KJJ81 revealed a total of 6121 gene models in the ATCC 36309 genome (Additional file 14: Figure S10). Initial nucleotide alignments of the ATCC 36309, KPH12, and KJJ81 genomes revealed that the ATCC 36309 genome is highly identical to the KPH12 genome, with 97.97% sequence identity. The identities of the ATCC 36309 genome in relation to subgenome A and subgenome B of KJJ81 were 97.85 and 89.97%, respectively, reflecting the fact that the ATCC 36309 genome is closely related to the KPH12 genome (Fig. 7a). The sequence of the ATCC 36309 rDNA unit was almost identical to that of KPH12 rDNA, with the exception of a deletion of three copies of the repeated sequence “TTAGCGAAAAAAAC” in the ETS2 region downstream of 25S. The positions of the putative centromere of ATCC 36309 and the telomere structures of ATCC 36309 were also highly identical to those observed in the KPH12 genome (Additional file 15: Figure S11). *S. fibuligera* ATCC 36309 possessed the α-pheromone-encoding *MFα*2 (306-bp ORF), but not the other *MFα1* gene, as observed in the KPH12 genome and KJJ81 subgenome A (Additional file 9: Figure S8b).

Quite interestingly, despite such high identity between the ATCC 36309 and KPH12 genomes, the synteny analysis of the ATCC 3609 and the KPH12 genomes revealed the presence of reciprocal translocation between chromosomes 3 and 5 (Fig. 7a). An interchromosomal translocation was also detected in the synteny analysis between the ATCC 36309 genome and KJJ81 subgenome A, but not between the ATCC 36309 genome and KJJ81 subgenome B. These observations indicated that while the original synteny was preserved without major rearrangement in the ATCC 36309 genome and KJJ81 subgenome B, a reciprocal translocation occurred between chromosomes 3 and 5 in the KPH12 genome (Fig. 7b). Thus, it is highly likely that the reciprocal translocation between chromosomes 3 and 5 occurred in the KPH12 genome before the KJJ81 genome was generated via hybrid formation.

There were several other interesting differences observed in the ATCC 36309 genome compared with the KPH12 and KJJ81 genomes. One notable difference was the sizes of chromosomes 6 and 7. In the ATCC 36309 genome, chromosome 7 was slightly larger than chromosome 6, a finding that was reversed in the other *S. fibuligera* genomes (Additional file 2: Table S7). A notable difference between the ATCC 36309 and KPH12 genomes was the deletion of a ~20-kb fragment containing five genes encoding SAP6, abfC, HGT1, RTA1 and YRF1-2 at the subtelomeric region of chromosome 3 in the ATCC 36309 genome (Additional file 16: Figure S12). The ATCC 36309 genome preserved the *sfBGL2* gene, which is present in subgenome B of *S*. *fibuligera* KJJ81, but absent in subgenome A of KJJ81 and the KPH12 genome (Additional file 2: Table S9). These findings indicate that the common ancestor of *S*. *fibuligera* contained the *BGL2* gene, but that it was lost in the KPH12 lineage. In addition, the loss of ~250 paralogous genes in the synteny relationship between the ATCC 36309 and KPH12 genomes reflects the fact that sequence divergence occurred during growth adaptation to different environments, such as *Nuruk* in Korea and chalky rye bread in Germany.

## Discussion

The widespread WG sequencing of yeast species in the last decade has provided new insights into the biodiversity, population structure, phylogeography, and evolutionary history of fungal populations [58]. However, most genomics studies have been conducted in *S. cerevisiae* and closely related yeast species, and high-quality whole genomes for other yeast species, such as members of the early-diverging *Saccharomycotina,* are therefore quite scarce. In the present study, we performed the WG de novo sequencing and complete assembly of two *S. fibuligera* isolates from wheat-based *Nuruk* in Korea, *S. fibuligera* KPH12 and KJJ81, and an isolate from chalky rye bread in Germany, *S. fibuligera* ATCC 36309. Comparative genomic analysis, based on the completely assembled *S. fibuligera* genomes from telomere to telomere, revealed the unique genome structure and evolutionary history of *S. fibuligera* and its interspecies hybrid. The *S. fibuligera* KJJ81 hybrid genome is composed of subgenomes A and B, which are derived from two progenitors, *S. fibuligera* KPH12 and a closely related (sub)species. The origin of the parent strain for subgenome B in the *S. fibuligera* KJJ81 hybrid isolate remains unknown, due to the lack of close sequence similarity among existing yeast species other than *S. fibuligera*.

The sequence divergence observed between both subgenomes A and B (10.84% at the nucleotide level between syntenic regions) was equivalent to the divergence described between the genomes of *S. cerevisiae* and *S. paradoxus*, two distinct species of the genus *Saccharomyces* [59] (Additional file 17: Figure S13). A plausible hypothesis for the formation of this architecture is believed to involve the hybridization of two genomes differing by approximately 10% via inter(sub)species mating. The highly conserved synteny over the whole genome in the *S. fibuligera* KJJ81 hybrid genome strongly indicates that a recent hybridization event occurred. Analysis of the synteny relationships of each pair of genes between subgenomes A and B of KJJ81 revealed that several subgenome A-specific single-copy genes were also present in both the KPH12 and ATCC 36309 genomes (Additional file 2: Table S8), indicating that these genes might have been lost from subgenome B. Due to the lack of information on *S. fibuligera* isolates belonging to the subgenome B lineage, whether the gene loss observed in subgenome B had occurred after or before the hybridization event cannot be determined. In contrast, a few genes, such as *COX7*, *BGL2*, *COX17*, *LSB5*, and *SATL1*, were present as intact ORFs in KJJ81 subgenome B, but present as truncated or altered spliced forms in both KJJ81 subgenome A and the KPH12 genome, indicating that inactivation of these genes had occurred in the subgenome A lineage before hybridization (Additional file 2: Table S9). The essentiality of rDNA for cell growth suggests a relatively recent loss of these rDNA clusters in the hybrid isolate KJJ81. Therefore, the *S. fibuligera* KJJ81 hybrid genome reflects the very early stage of genome stabilization, which preserves most of the synteny relationship between two subgenomes, but involves recent unilateral loss of the rDNA cluster and a few genes in subgenome B (Fig. 7c). The hybrid genome of *S. fibuligera* KJJ81 offers an interesting snapshot of the genomic evolutionary events that occurred after inter(sub)specific hybridization.

When we compared the growth phenotypes of the *S. fibuligera* KJJ81, KPH12, and ATCC 36309, the two isolates from *Nuruk* showed higher thermotolerance than the isolate from chalky rye bread in Germany. Moreover, the hybrid KJJ81 appeared more adapted to higher temperatures (Additional file 18: Figure S14a) than KPH12, thus exhibiting a higher survival potential during *Nuruk* fermentation, which is typically conducted at a continuous temperature range of 30–45 °C [60]. Therefore, such temperature variation during *Nuruk* fermentation might generate certain selective pressures on particular communities with higher thermotolerance over others. In addition, KJJ81 exhibited more adaptive growth in the presence of inorganic sulfur as the only S source compared to KPH12 (Additional file 18: Figure S14b), indicating that some advantageous properties were generated by hybrid formation.

The *S. fibuligera* genome with high-quality annotation establishes an important foundation for making functional inference of *S. fibuligera* in the digestion of fermentation mash. In analyzing the genomes of KJJ81 and KPH12, we unexpectedly discovered numerous genes for extracellular hydrolytic enzymes, such as amylase, β-glucosidase, cellulase, and acidic protease, involved in saccharification and proteolysis (Additional file 13). During cultivation on *Nuruk* or bread, *S. fibuligera* grows on the surface of whey or barley, where amino acids and sugars are initially deficient. The need for *S. fibuligera* to effectively obtain access to external carbon and nitrogen sources thorough the degradation of proteins and starches appears consistent with the observed expansion of hydrolytic enzymes in the *S. fibuligera* genome, strongly indicating that *S. fibuligera* is highly saccharolytic and proteolytic, resulting in its dominant appearance as a major yeast species in Asian traditional alcoholic starters made of various grains. It is noteworthy that the expression patterns of these extracellular hydrolytic enzymes generally appeared to be induced under nutrient-limited conditions, such as cultivation in low-glucose or B-minimal medium (Figs. 5, 6). The *S. fibuligera* genes that have been newly discovered through genome research in this study are expected to be applicable to the production of novel valuable enzymes and chemicals. To obtain information on the substrate specificity and functional features of the new *S. fibuligera* enzymes, secretory expression and purification of these enzymes as recombinant proteins in a heterologous host system are currently underway for subsequent biochemical characterization, particularly focusing on those enzymes associated with polysaccharide degradation including novel β-glucosidases, PMOs, and abfC.

Intensive research efforts have recently been made in searching for and genome sequencing of microorganisms that secrete hydrolytic enzymes with high potential for applications in sectors such as the food, detergent, laundry, textile, baking, and biofuel industries [61, 62]. The distinctive genomic inventory of *S. fibuligera*, enriched in genes for extracellular hydrolytic enzymes, further supports the high potential of this amylolytic yeast to serve as an economical host for the production of industrial enzymes and bioproducts from renewable resources. However, our RNA-Seq analysis showed that the production of these enzymes is subject to catabolite repression at transcription levels by glucose or other available nutrients. Therefore, to more efficiently exploit *S. fibuligera* as a cell factory for bioconversion, it is necessary to elucidate the gene regulatory networks underlying its metabolism with industrial potential and to systematically modulate the expression of relevant genes, such as transcription factor genes, in this yeast. The complete WG sequence of *S. fibuligera* with high-quality annotation will facilitate the development of new tools for genetic manipulation and allow the application of systems biology approaches to the identification of metabolic targets in engineering *S. fibuligera* strains for the production of biofuels and chemicals based on bioconversion of starchy or cellulosic biomass.

The genus *Saccharomycopsis,* in the phylum *Ascomycota,* was initially described by Schiönning (1903) with only the species *Saccharomycopsis capsularis*, but it has currently been expanded to at least 17 species [63]. As the first gapless genome assembly in the genus *Saccharomycopsis*, the genome information obtained for *S. fibuligera* and its hybrid in the present study is also expected to provide a useful basis for the elucidation and documentation of evolutionary consequences within fungal populations. Phylogenetic position analysis based on our comparative analysis of *S. fibuligera* genome indicated the *Saccharomycopsis* clade as an early divergent of the subphylum *Saccharomycotina*. Interestingly, the majority of the species of the genus *Saccharomycopsis* are predacious yeasts that are able to penetrate and kill various yeast prey [46]. Some members of the genus *Saccharomycopsis* have drawn increasing attention due to exhibiting unique physiological characteristics that are useful for various biotechnological applications, such as *Saccharomycopsis* fermentation filtrate as a component of cosmetics [64] and high hydrolytic activity for the bioremediation of agricultural waste [20]. The *S. fibuligera* genome will serve as a useful basis for comparative genomics studies to investigate functional peculiarities specific to this yeast and its relative lineage within the *Saccharomycopsis* clade.

## Conclusions

The completely sequenced *S. fibuligera* genome with high-quality annotation and the RNA-Seq analysis, presented in this report, will greatly appeal to researchers with a broad spectrum of interests in both basic and applied areas of biological research. It establishes an important foundation for functional inference of *S. fibuligera* in the degradation of fermentation mash. The unique gene inventory of *S. fibuligera* provides insights into novel physiological activities with potent biotechnical applications, which requires further research to be elucidated. As a high-quality reference genome, it will elucidate biological peculiarities specific to this yeast and its relative lineage, facilitating various omics analyses to investigate metabolic pathways with biotechnological potential and their regulatory networks under culture conditions relevant to industrial processes. This information will also allow the implementation of new genetic manipulation platforms for *S. fibuligera,* applicable to the development of engineered yeast strains for simultaneous saccharification/fermentation processes, along with improved starter strains tailored for the production of rice wine with desired flavor profiles or an improved nutraceutical composition. Moreover, as the first report of a gapless WG assembled within the *Saccharomycopsis* genus with a high bioremediation capacity, the genomic information obtained for *S. fibuligera* and its interspecies hybrid strain provides an important foundation for comparative biological analyses, representing a useful basis for the delineation and documentation of evolutionary consequences and divergence in fungal populations.

## Methods

### Strains, media, and cultivation conditions

*Saccharomycopsis fibuligera* KJJ81 and KPH12 strains were isolated from *Nuruk* samples collected from Jeju (KJJ81) and Pohang (KPH12), respectively. The 5.8S rDNA sequences flanked by ITS regions 1 and 2 [65] were amplified using primers ITS-1 (5′-TCC GTA GGT GAA CCT GCGG-3′) and ITS-4 (5′-TCC TCC GCT TAT TGA TAT GC-3′) and were analyzed using ISHAM ITS DB (http://its.mycologylab.org). *Saccharomycopsis fibuligera* ATCC 36309 (KCTC 7806, NRRL Y-2388), an isolate from chalky rye bread in Germany, was used as a type strain of *S. fibuligera*. Yeast cells were cultured using YP (1% yeast extract, 2% Bacto peptone) medium containing 2% glucose (D2) or 0.1% glucose (D0.1). For sulfur assimilation analysis, solid sulfur-free B medium (synthetic medium with 2% glucose without any sulfur source) [47] was made with 1% agarose instead of agar.

### Genome sequencing and assembly

For WG sequencing of *S. fibuligera* KPH12 and KJJ81, long, short, and long-mated pair reads were produced using PacBio RS II, TruSeq Synthetic Long Reads, and Illumina HiSeq 2500 sequencing technologies (Additional file 2: Table S1). WG de novo assembly for KPH12 and KJJ81 was performed via a hybrid approach (Additional file 1: Figure S1, Additional file 2: Table S2). The quality of the assemblies was assessed by aligning short-insert reads to the two assemblies (Additional file 2: Table S3). Quality assessment of genome assemblies was performed by comparing between the resulting scaffolds and the TSLR assembly using QUAST [66] (Additional file 2: Table S4). Moreover, the mapping rates of short reads to the scaffolds and their insert size distribution were examined. For WG de novo assembly of ATCC 36309, long-read SMRT and Illumina (500-bp short-insert reads) sequence data were produced, and scaffolds were assembled based on KPH12 assembly data using the above-mentioned method.

### BioNano physical mapping

A DNA plug containing the *S. fibuligera* KPH12 genome was prepared using the CHEF Yeast Genomic DNA Plug Kit (Bio-Rad) with slightly modified conditions, treated with the nicking enzyme Nt.BspQI (New England BioLabs, NEB), labeled using a fluorescent nucleotide analog with the IrysPrep Reagent Kit (BioNano Genomics, Inc.), and then loaded onto the nanochannel array of a BioNano Genomics IrysChip via DNA electrophoresis.

### RNA-Seq analysis

For RNA-Seq analysis, yeast cells cultivated overnight were initially inoculated at an OD_600_ (optical density at 600 nm)  of 0.2 in YP medium containing 2%, 0.1% glucose, 2% starch, and B medium and were grown to early logarithmic phase (OD_600_  =  0.5) at 37 °C. Yeast cells were collected by filtration using a 10-µm polyvinylidene difluoride membrane. Total RNA was isolated via a physical cell breaking method [67], and RNA-Seq was then performed with an Illumina HiSeq 2500 instrument. RNA-Seq libraries were prepared using a TruSeq RNA Sample Prep Kit (Illumina, Inc., San Diego, CA, USA). After qPCR validation using a KAPA library quantification kit (KAPA Biosystems, South Africa), libraries were subjected to paired-end sequencing with a 100-bp read length using an Illumina HiSeq 2500 platform, with ≥38 million reads per library. For the validation of genome annotation based on RNA-Seq data, clean reads with average quality scores of more than Q30 for all libraries were aligned to the KPH12 and KJJ81 genomes using the STAR spliced read aligner [68] with a set of gene model annotations. Total counts of read fragments aligned to protein-coding genes were obtained using an HTSeq [69] and were used as a basis for the quantification of gene expression. Differential expression analysis between sample groups of interest (i.e., D0.1 vs. D2, B vs. D2) was performed using the TCC package [70].

### Genome annotation

Gene models were predicted by combining evidence from transcriptome and protein sequence alignments with ab initio prediction on the basis of repeat-masked genome sequences. GeneMark-ET [71] was used to perform iterative training and to generate initial gene structures with RNA-Seq data information. AUGUSTUS [72] was further used to perform de novo prediction with gene models trained by GeneMark-ET, with exon–intron boundary information predicted by transcriptome and protein sequence alignments. TopHat [73] was used for RNA-Seq alignment, and Exonerate [74] was used for protein sequence alignment with subphylum *Saccharomycotina* sequences. In addition, homologous genes between KPH12 and KJJ81 were searched using TBLASTN [75], with an *E*-cutoff value of 1*E*-10, and MCScanX [76] to identify missing genes through de novo prediction. For functional annotation, genes were searched against the UniProt, NCBI non-redundant (NR), and relative fungi RefSeq (i.e., *S. cerevisiae*, *C. glabrata*, *A. oryzae*, and *S. pombe*) databases using BLASTP [75] with an *E*-cutoff value of 1*E*-10. Protein domains were also searched using InterProScan [77]. Gene ontology (GO) and Kyoto Encyclopedia of Genes and Genomes (KEGG) pathway annotations were performed using Blast2GO [78].

The tRNA genes were identified with tRNAscan-SE [79] using default parameters. The rRNAs, collected from the NCBI NT database, were compared with the genomes using BLASTN [75]. Other non-coding RNAs, including miRNA and snRNA, were identified using INFERNAL [80] via comparison with the Rfam. Repetitive DNA sequences, including retrotransposons, DNA transposons, microsatellites, and other repeats, were screened using RepeatMasker [81], which was developed for de novo repeat family identification and modeling. The repeat-masked scaffolds were used for gene prediction as described above. Mitochondrial genome annotation was performed using MFannot (http://megasun.bch.umontreal.ca/cgi-bin/mfannot/mfannotInterface.pl) with the correction of ORF structures. Synteny blocks between two genomes (i.e., KPH12 vs. KJJ81, KJJ81 subgenome A vs. KJJ81 subgenome B, KPH12 vs. ATCC 36309, and KJJ81 vs. ATCC 36309) were identified with SyMAP [82].

### Phylogeny and gene family analysis

For the construction of a genome-based phylogeny, protein sequences of KPH12 were searched against those of 12 species, including *A. gossypii*, *K. lactis*, *C. glabrata*, *S. cerevisiae*, *C. lusitaniae*, *C. tropicalis*, *D. hansenii*, *Y. lipolytica*, *A. fumigatus*, *A. oryzae*, and *S. pombe*, which were downloaded from Ensembl Fungi (http://fungi.ensembl.org/index.html). The search was performed using reciprocal BLAST, a common computational method to predict orthologous genes. The highest-scoring genes were taken with the following: (1) ≥50% sequence identity and (2) 10% deviation in compared sequence length. A total of 55 orthologous genes (Additional file 10) were finally selected from among 12 species, and a single concatenating sequence per species was built. The resulting sequences were multiple-aligned using ClustalW [83]. A phylogenetic tree was constructed using MEGA6 [39] via the neighbor-joining method, with bootstrap values for 1000 replicates. Orthologous gene families were assigned to the 12 species against the Pfam database using HMMPfam.

### Accession numbers

The assembled sequences for the *S. fibuligera* KJJ81 and KPH12 chromosomal genomes were deposited in the GenBank database under the accession nos. CP012809–CP012822 and CP012823–CP012829, respectively (nuclear genome). The assembled genome sequence for *S. fibuligera* ATCC 36309 was deposited in the GenBank database under the accession nos. CP015978–CP015984 (nuclear genome). The raw RNA-Seq data were deposited in the BioSample database of NCBI. Accession nos. SAMN05180633, SAMN05180634 (KJJ81 on B medium); SAMN05180635, SAMN05180636 (KJJ81 on YP containing glucose 0.1%); SAMN05180637, SAMN05180638 (KJJ81 on YP containing glucose 2%); SAMN05180639, SAMN05180640 (KPH12 on B medium); SAMN05180641, SAMN05180642 (KPH12 on YP containing glucose 0.1%); SAMN05180637, SAMN05180638 (KPH12 on YP containing glucose 2%).

## Additional files

**Additional file 1: Figure S1.** Dimorphic growth analysis of *S. fibuligera* KPH12 and KJJ81.

**Additional file 2: Table S1.** Sequencing summary of the *S. fibuligera* KPH12 and KJJ81 genomes. **Table S2.** Summary of the de novo assembly of the *S. fibuligera* KPH12 and KJJ81 genomes. **Table S3.** Alignment statistics for short-insert reads aligned to the scaffolds of the *S. fibuligera* KPH12 and KJJ81 genomes. **Table S4.** Assessment of the quality of de novo assembly through comparison between the TSLR assembly and the draft genome of *S. fibuligera* KPH12 and KJJ81. **Table S5.** Functional annotation of predicted protein-coding genes in the *S. fibuligera* KPH12 and KJJ81 genomes. **Table S6.** Protein-coding genes annotated in the *S. fibuligera* mitochondrial genome. **Table S7.** Comparison of scaffold length and GC percentage among the *S. fibuligera* KPH12, KJJ81, and ATCC 36309 genomes. **Table S8.** Subgenome A-specific single genes of *S. fibuligera* KJJ81. **Table S9.** Subgenome B-specific single genes of *S. fibuligera* KJJ81.

**Additional file 3: Figure S2.** Methods employed in this study for the WG de novo sequencing and assembly of *S. fibuligera* KPH12 and KJJ81.

**Additional file 4: Figure S3.** Genome coverage and sequencing depth of short insert reads aligned to the scaffolds of *S. fibuligera.*

**Additional file 5: Figure S4.** Sequence assembly data anchored to the BioNano physical map of *S. fibuligera* KPH12.

**Additional file 6: Figure S5.** Mitochondrial genomes of *S. fibuligera* KPH12 and KJJ81.

**Additional file 7: Figure S6.** The putative centromere regions on the *S. fibuligera* KPH12 and KJJ81 genomes.

**Additional file 8: Figure S7.** Identification of repeat sequences at both ends of the *S. fibuligera* KPH12 and KJJ81 chromosomes.

**Additional file 9: Figure S8.** Comparative analysis of mating type locus (*MAT*) organization and mating-type hormone genes between *S. fibuligera* subgenomes A and B.

**Additional file 10.** Orthologous genes used for genome-based phylogenetic analysis.

**Additional file 11.** Gene symbols, annotations and expression abundance data for *S. fibuligera* genes involved in carbon and sulfur metabolism.

**Additional file 12: Figure S9.** Comparative analysis of growth, glucose utilization, and ethanol and glycerol production between two *S. fibuligera* isolates and *S. cerevisiae* in the presence of different concentrations of glucose.

**Additional file 13.** Gene symbols, annotations and expression abundance data for *S. fibuligera* genes involved in the degradation of cellulose, starch, and cell wall polysaccharides, and the genes coding for acidic protease.

**Additional file 14: Figure S10.** Summary of the annotation process for the *S. fibuligera* ATCC 36309 genome.

**Additional file 15: Figure S11.** Identification of putative centromeres and telomere regions of *S. fibuliger*a ATCC 36309 chromosomes.

**Additional file 16: Figure S12.** Deletion of a ~20 kb fragment containing five genes encoding *SAP6*, abfC, *HGT1*, *RTA1* and YRF1-2 in the subtelomeric region of chromosome 3 of *S. fibuligera* ATCC 36309.

**Additional file 17: Figure S13.** Sequence divergence between subgenomes in hybrid yeast species.

**Additional file 18: Figure S14.** Comparative growth analysis of *S. fibuligera* isolates at different temperatures and on different sulfur sources.

## Abbreviations

ITS: internal transcribed spacer
WG: whole genome
TSLR: TruSeq synthethic long reads
rDNA: ribosomal DNA
mtDNA: mitochondrial genome
NTS: nontranscribed spacers
LTR: long terminal repeat
TCA: tricarboxylic acid cycle
Glc: glucose
G6P: glucose 6-phosphate
F6P: fructose 6-phosphate
F1,6Bp: fructose 1,6-bisphosphate
DHAP: dihydroxyacetone phosphate
G3P: glyceraldehyde 3-phosphate
1,3Bpg: 1,3-bisphosphoglycerate
3Pg: 3-phosphoglycerate
2Pg: 2-phosphoglycerate
Pep: phosphoenolpyruvate
AcCoA: acetyl-CoA
CoA: coenzyme A
Pyr: pyruvate
Glycerol-p: glycerol 3-phosphate
OAA: oxaloacetic acid
AKG: alpha-ketoglutarate
T6P: trehalose 6-phosphate
6-Pgdl: 6-phosphogluconolactone
6PG: 6-phosphogluconate
Ru5P: ribulose-5-phosphate
R5P: ribose-5-phosphate
Xu5P: xylulose 5-phosphate
S7P: sedoheptulose-7-phosphate
E4P: erythrose-4-phosphate
Homo-ser: homoserine
OAH: *O*-acetyl homoserine
Homo-cys: homocysteine
Met: methionine
AdoMet: *S*-adenosylmethionine
AdoHC: *S*-adenosylhomocysteine
Ser: serine
OAS: *O*-acetyl serine
Cys: cysteine
GSH: glutathione
GPI: glycosylphosphatidylinositol
