# Supplementary material for: Whole-genome de novo sequencing, combined with RNA-Seq analysis, reveals unique genome and physiological features of the amylolytic yeast Saccharomycopsis fibuligera and its interspecies hybrid
Source: Biotechnol Biofuels. 2016 Nov 11;9:246. doi: 10.1186/s13068-016-0653-4 (PMC5106798; doi:10.1186/s13068-016-0653-4)
Supplement: Supplementary file 1 — Additional file 1: Figure S1. Dimorphic growth analysis of S. fibuligera KPH12 and KJJ81. [file 13068_2016_653_MOESM1_ESM.pdf]

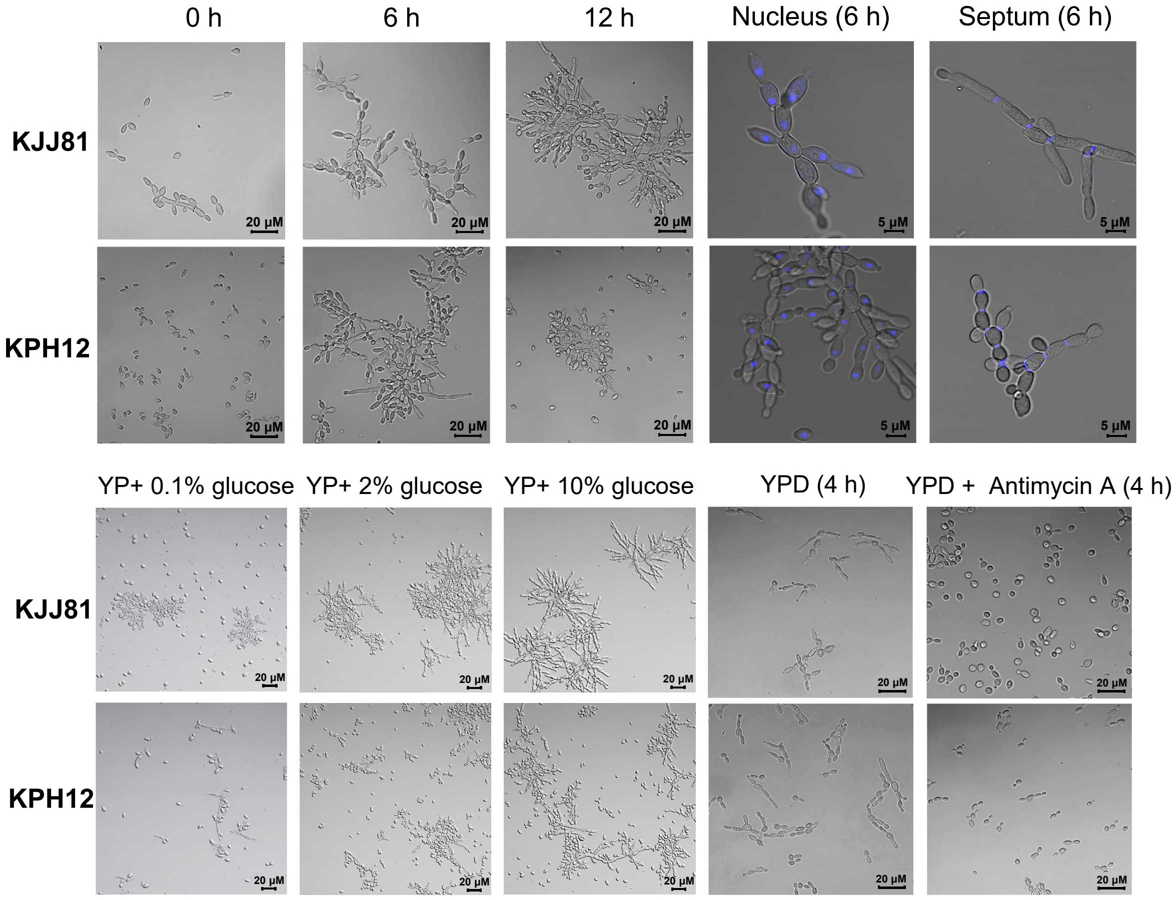

Figure S1. Dimorphic growth analysis of *S. fibuligera* KPH12 and KJJ81. Dimorphic growth, nucleus and septum staining analysis. Yeast-type cells were enriched via the filtration of overnight-cultivated cells and were used to inoculate liquid YPD medium at 37°C. The morphologies of *S. fibuligera* KPH12 and KJJ81 were analyzed by confocal microscopy (DIC) at the indicated culture times. Nuclear Hoechst staining and Calcofluor-white (CFW) staining of septa were analyzed using confocal microscopy.
