# Supplementary material for: Whole-genome de novo sequencing, combined with RNA-Seq analysis, reveals unique genome and physiological features of the amylolytic yeast Saccharomycopsis fibuligera and its interspecies hybrid
Source: Biotechnol Biofuels. 2016 Nov 11;9:246. doi: 10.1186/s13068-016-0653-4 (PMC5106798; doi:10.1186/s13068-016-0653-4)
Supplement: Supplementary file 2 — Additional file 2: Table S1. Sequencing summary of the S. fibuligera KPH12 and KJJ81 genomes. Table S2. Summary of the de novo assembly of the S. fibuligera KPH12 and KJJ81 genomes. Table S3. Alignment statistics for short-insert reads aligned to the scaffolds of the S. fibuligera KPH12 and KJJ81 genomes. Table S4. Assessment of the quality of de novo assembly through comparison between the TSLR assembly and the draft genome of S. fibuligera KPH12 and KJJ81. Table S5. Functional annotation of predicted protein-coding genes in the S. fibuligera KPH12 and KJJ81 genomes. Table S6. Protein-coding genes annotated in the S. fibuligera mitochondrial genome. Table S7. Comparison of scaffold length and GC percentage among the S. fibuligera KPH12, KJJ81, and ATCC 36309 genomes. Table S8. Subgenome A-specific single genes of S. fibuligera KJJ81. Table S9. Subgenome B-specific single genes of S. fibuligera KJJ81. [file 13068_2016_653_MOESM2_ESM.pdf]

Supplementary Tables

Supplementary Table 1. Sequencing summary of the *S. fibuligera* KPH12 and KJJ81 genomes

	KPH12	KJJ81
Short-insert paired-end reads (500 bp[1])		
Raw reads (No.)	24,132,834	22,897,290
Total bases (coverage, X)	122.91	59.34
Q20 bases (%)	88.86	86.23
Long-mate paired-end reads (15 kb[1])		
Raw reads (No.)	44,904,808	54,748,720
Total bases (coverage, X)	228.91	141.89
Q20 bases (%)	87.14	87.75
TSLRs		
Long reads (No.)	94,757	80,922
Average length of reads (bp)	4,806	5,016
Total bases (coverage, X)	23.19	10.52
Long SMRT sequencing reads		
Long reads (No.)	207,691	388,982
Average length of leads (bp)	8,955	9,226
Total bases (coverage, X)	94.73	94.44

[1]Insert size of the sequencing library

Supplementary Table 2. Summary of the *de novo* assembly of the *S. fibuligera* KPH12 and KJJ81 genomes

	KPH12	KJJ81
Contig assembly by long reads[1]		
Contig number	18	45
Total length (bp)	19,193,493	37,774,778
N50	3,000,789	1,903,357
N90	1,353,387	721,347
Longest	4,132,216	3,922,141
Scaffolding and gap filling		
Scaffold number	7	14
Total length (bp)	19,567,216	38,516,460

[1]Contig assembly using long SMRT sequencing reads

Supplementary Table 3. Alignment statistics for short-insert reads aligned to the scaffolds of the *S. fibuligera* KPH12 and KJJ81 genomes

	Library	Raw reads (No.)	Mapped reads (No.)	Mapped reads (%)	Mapped paired-end reads (No.)	Mapped paired-end reads (%)
KPH12	PE	24,132,834	20,359,623	84.36	19,676,582	81.53
	MP	15,453,530	14,499,800	93.83	12,169,148	78.75
KJJ81	PE	22,897,290	20,553,001	89.76	18,153,674	79.28
	MP	14,830,464	13,610,930	91.78	7,593,382	51.2

PE: Paired end, MP: Mating pair

Supplementary Table 4. Assessment of the quality of *de novo* assembly through comparison between the TSLR assembly and the draft genome of *S. fibuligera* KPH12 and KJJ81

Metric	Value	
	KPH12	KJJ81
No. of contigs (≥ 1 kb)	292	1,025
Total length (bp)	19,483,087	37,860,684
Physical coverage (%)	99.62	98.37
Longest contig length (bp)	614,411	446,381
N50 contig length (bp)	157,627	71,542
N90 contig length (bp)	43,980	19,473
Contig GC content (%)	38.07	38.41
Genome fraction (%)	96.368	96.559
Duplication ratio	1.031	1.019
NA50	156,180	70,317
LA50	42	170
Mismatches per 100 kb	6.05	12.2
InDels per 100 kb	15.45	14.74
Ns per 100 kb	0.05	0.05
Fully unaligned contigs (No.)	1	4
Fully unaligned length (bp)	1,002	2,373
Partially unaligned contigs (No.)	11	6
Fully unaligned length (bp)	89,551	74,561

Supplementary Table 5. Functional annotation of predicted protein-coding genes in the *S. fibuligera* KPH12 and KJJ81 genomes

	KPH12		KJJ81	
	No. of genes	Percentage (%)	No. of genes	Percentage (%)
Predicted genes	6,155		12,185	
Annotated	5,435	88.30	10,810	88.72
SwissProt	4,767	77.45	9,464	77.67
InterPro	4,998	81.20	9,959	81.73
NCBI NR	5,222	84.84	10,367	85.08
GO	3,956	64.27	7,885	64.71
KEGG	993	16.13	2,751	22.58
Unannotated	720	11.70	1,375	11.28

Supplementary Table 6. Protein-coding genes annotated in the *S. fibuligera* mitochondrial genome

Gene	KJJ81	KPH12	Gene	KJJ81	KPH12
Complex I			tRNAs		
nad1*	+a	+a	trnR(acg)	+a	+a
nad2	+	+	trnS(gct)	+a	+a
nad3	+	+	trnD(gtc)	+a	+a
nad4	+	+	trnQ(ttg)	+a	+a
nad4L	+	+	trnL(taa)	+a	+a
nad5*	+	+	trnR(tct)	+a	+a
nad6	+a	+a	trnV(tac)	+a	+a
Complex III			trnC(gca)	+a	+a
cob*	+	+	trnY(gta)	+a	+a
Complex IV			trnM(cat)	+b	+b
cox1*	+	+	trnA(tgc)	+a	+a
cox2	+	+	trnN(gtt)	+a	+a
cox3	+	+	trnT(tgt)	+	+
Complex V			trnE(ttc)	+	+
atp6	+	+	trnP(tgg)	+	+
atp8	+	+	trnT(tag)	+	+
atp9	+a	+a	trnK(ttt)	+	+
Ribosomal RNAs			trnF(gaa)	+	+
rnl	+a	+a	trnH(gtg)	+	+
rns	+	+	trnS(tga)	+	+
Ribonuclaease			trnI(gat)	+	+
rnpB	+	+	trnW(tca)	+	+
Other ORF			trnG(tcc)	+	+
orf1057	+a	+			
orf345	+	+			
orf856	-	+			

+ : present, - : absent, * : include intron, a : two copies, b : four copies

Supplementary Table 7. Comparison of scaffold length and GC percentage among the *S. fibuligera* KPH12, KJJ81, and ATCC 36309 genomes

Chr.	KJJ81A		KJJ81B		KPH12		ATCC 36309	
Number	bp	%GC	bp	%GC	bp	%GC	bp	%GC
1	4,911,352	38.63	4,344,079	38.92	4,897,519	38.63	4,909,981	38.69
2	4,155,118	37.93	4,044,695	38.56	4,145,645	37.96	4,155,432	37.98
3	3,027,211	38.14	2,646,735	38.63	3,010,680	38.13	2,682,731	37.97
4	2,704,859	38.13	2,652,149	38.96	2,681,439	38.06	2,685,930	38.02
5	2,120,103	37.99	2,381,737	38.92	2,105,831	38.02	2,406,875	38.18
6	1,449,650	38.63	1,410,702	39.19	1,462,644	38.67	1,400,908	38.36
7	1,364,239	38.31	1,345,124	39.37	1,359,543	38.29	1,411,284	38.48
total	19,732,532	38.24	18,825,221	38.86	19,663,301	38.25	19,653,141	38.25

Supplementary Table 8. Subgenome A-specific single genes of *S. fibuligera* KJJ81

Locus taq	Annotation	Description	KPH12	ATCC 36309
KJJ81A1G091600	*SPO22*	Sporulation-specific protein 22	KPH12A1G091600	KCTCA1G102950
KJJ81A1G142200	vioD	Capreomycidine synthase	KPH12A1G142000	KCTCA1G162100
KJJ81A2G021000	YBL028C	UPF0642 protein YBL028C	KPH12A2G020900	KCTCA2G020500
KJJ81A2G049700	SPBC2G2.13c	Deoxycytidylate deaminase	KPH12A2G049400	KCTCA2G052700
KJJ81A3G026400	*CIS3*	Cell wall mannoprotein CIS3	KPH12A3G026200	KCTCA3G032250
KJJ81A3G090600	*SAP6*	Candidapepsin-6	KPH12A3G089500	-
KJJ81A4G003400	YDR286C	Glutaredoxin-like protein YDR286C	KPH12A4G002900	KCTCA4G002900
KJJ81A5G063400	*KNH1*	Cell wall synthesis protein KNH1	KPH12A5G063500	KCTCA3G090050
KJJ81A6G019900	*SOH1*	Mediator of RNA polymerase II transcription subunit 31	KPH12A6G019600	KCTCA6G022750
KJJ81A6G044400	*GLA1*	Glycosyl hydrolases family 15	KPH12A6G043900	KCTCA6G049450
KJJ81A7G009100	*MSH5*	MutS protein homolog 5	KPH12A7G009200	KCTCA7G009300
KJJ81A7G024200	qorB	Quinone oxidoreductase 2	KPH12A7G024100	KCTCA7G024300
KJJ81A7G030400	SPAC869.02c	Flavohemoprotein	KPH12A7G030200	KCTCA7G030200

- : not present

Supplementary Table 9. Subgenome B-specific single genes of *S. fibuligera* KJJ81

Locus taq	Annotation	description	KPH12	ATCC 36309
KJJ81B1G099200	*COX7*	Cytochrome c oxidase subunit 7	-	-
KJJ81B1G102300	*BGL2*	Beta-glucosidase 2	-	KCTCA1G116900
KJJ81B2G015800	*COX17*	Cytochrome c oxidase copper chaperone	-	-
KJJ81B5G075500	*LSB5*	LAS seventeen-binding protein 5	-	-
KJJ81B7G033100	*SATL1*	Spermidine/spermine N(1)-acetyltransferase-like protein 1	-	-

- : not present
