# Supplementary material for: Whole-genome de novo sequencing, combined with RNA-Seq analysis, reveals unique genome and physiological features of the amylolytic yeast Saccharomycopsis fibuligera and its interspecies hybrid
Source: Biotechnol Biofuels. 2016 Nov 11;9:246. doi: 10.1186/s13068-016-0653-4 (PMC5106798; doi:10.1186/s13068-016-0653-4)
Supplement: Supplementary file 3 — Additional file 3: Figure S2. Methods employed in this study for the WG de novo sequencing and assembly of S. fibuligera KPH12 and KJJ81. [file 13068_2016_653_MOESM3_ESM.pdf]

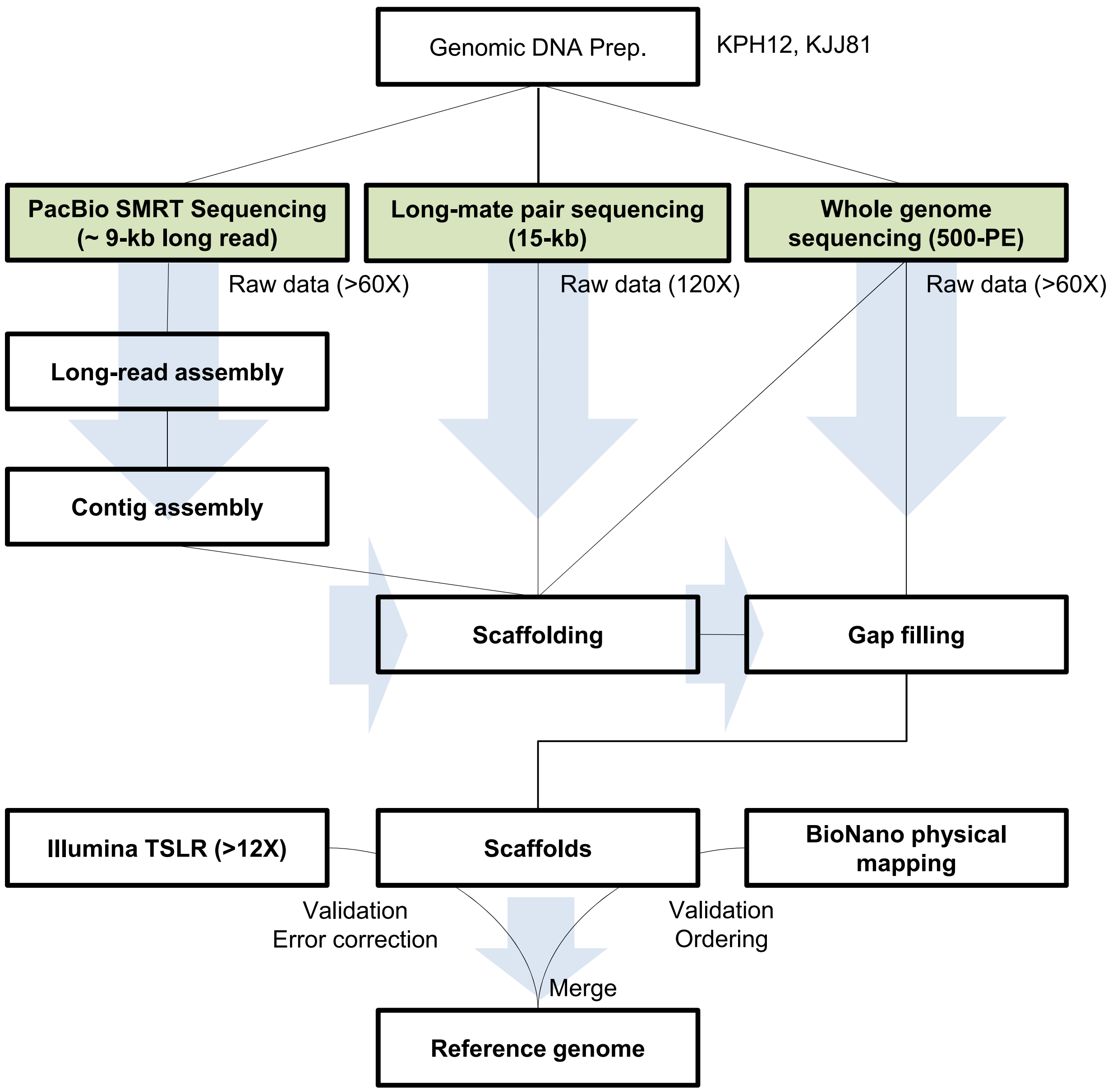

Figure S2. Methods employed in this study for the WG *de novo* sequencing and assembly of *S. fibuligera* KPH12 and KJJ81. A total of ≥ 94x long reads with an average length of 9 kb were produced using PacBio RS II technology (Pacific Biosciences). In addition, a TruSeq Synthetic Long Reads (TSLR) sequencing library (Illumina) was also constructed and then sequenced with 23x and 10x coverage for KPH12 and KJJ81, respectively. Next, ≥ 86x short insert reads (a 500-bp library) and ≥ 87x long-mated pair reads (a 15-kb library) were produced using Illumina HiSeq2500 sequencing technology with 100-bp paired-end sequencing following an Illumina genomic DNA library construction protocol. For WG sequencing of *S. fibuligera* ATCC 36309, a total of ≥ 94x long reads with an average length of 9 kb were produced using PacBio technology, and ≥ 60x short insert reads (a 500-bp library) were produced using Illumina HiSeq. Long SMRT sequencing reads were assembled using HGAP3, and 15-kb mate-pair and 500-bp short insert reads were then used to construct scaffolds with SSPACE software [1]. Gaps were filled with the short read data using GapCloser [2].

Reference

1. Boetzer M, Henkel CV, Jansen HJ, Butler D, Pirovano W. Scaffolding pre-assembled contigs using SSPACE. Bioinformatics. 2011; 27:578-579.

2. Luo RB, Liu BH, Xie YL, Li ZY, Huang WH, Yuan JY, et al. SOAPdenovo2: an empirically improved memory-efficient short-read de novo assembler. Gigascience. 2012; 1:18.
