# Supplementary material for: Whole-genome de novo sequencing, combined with RNA-Seq analysis, reveals unique genome and physiological features of the amylolytic yeast Saccharomycopsis fibuligera and its interspecies hybrid
Source: Biotechnol Biofuels. 2016 Nov 11;9:246. doi: 10.1186/s13068-016-0653-4 (PMC5106798; doi:10.1186/s13068-016-0653-4)
Supplement: Supplementary file 4 — Additional file 4: Figure S3. Genome coverage and sequencing depth of short insert reads aligned to the scaffolds of S. fibuligera. [file 13068_2016_653_MOESM4_ESM.pdf]

a.

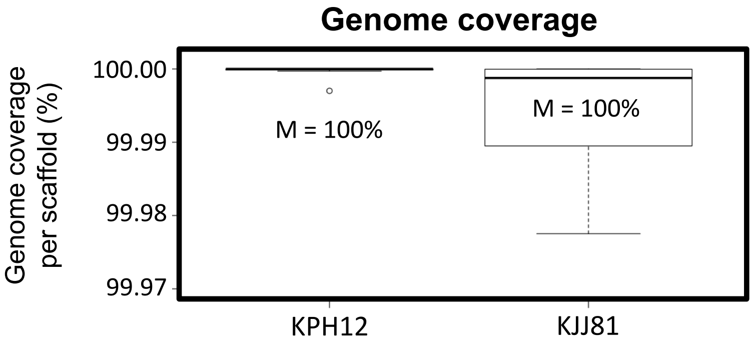

b.

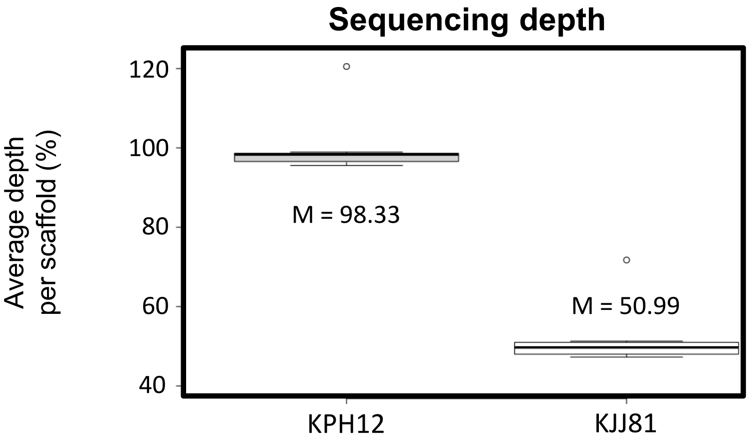

Figure S3. Genome coverage **(a)** and sequencing depth **(b)** of short insert reads aligned to the scaffolds of *S. fibuligera*.
