# Supplementary material for: Whole-genome de novo sequencing, combined with RNA-Seq analysis, reveals unique genome and physiological features of the amylolytic yeast Saccharomycopsis fibuligera and its interspecies hybrid
Source: Biotechnol Biofuels. 2016 Nov 11;9:246. doi: 10.1186/s13068-016-0653-4 (PMC5106798; doi:10.1186/s13068-016-0653-4)
Supplement: Supplementary file 5 — Additional file 5: Figure S4. Sequence assembly data anchored to the BioNano physical map of S. fibuligera KPH12. [file 13068_2016_653_MOESM5_ESM.pdf]

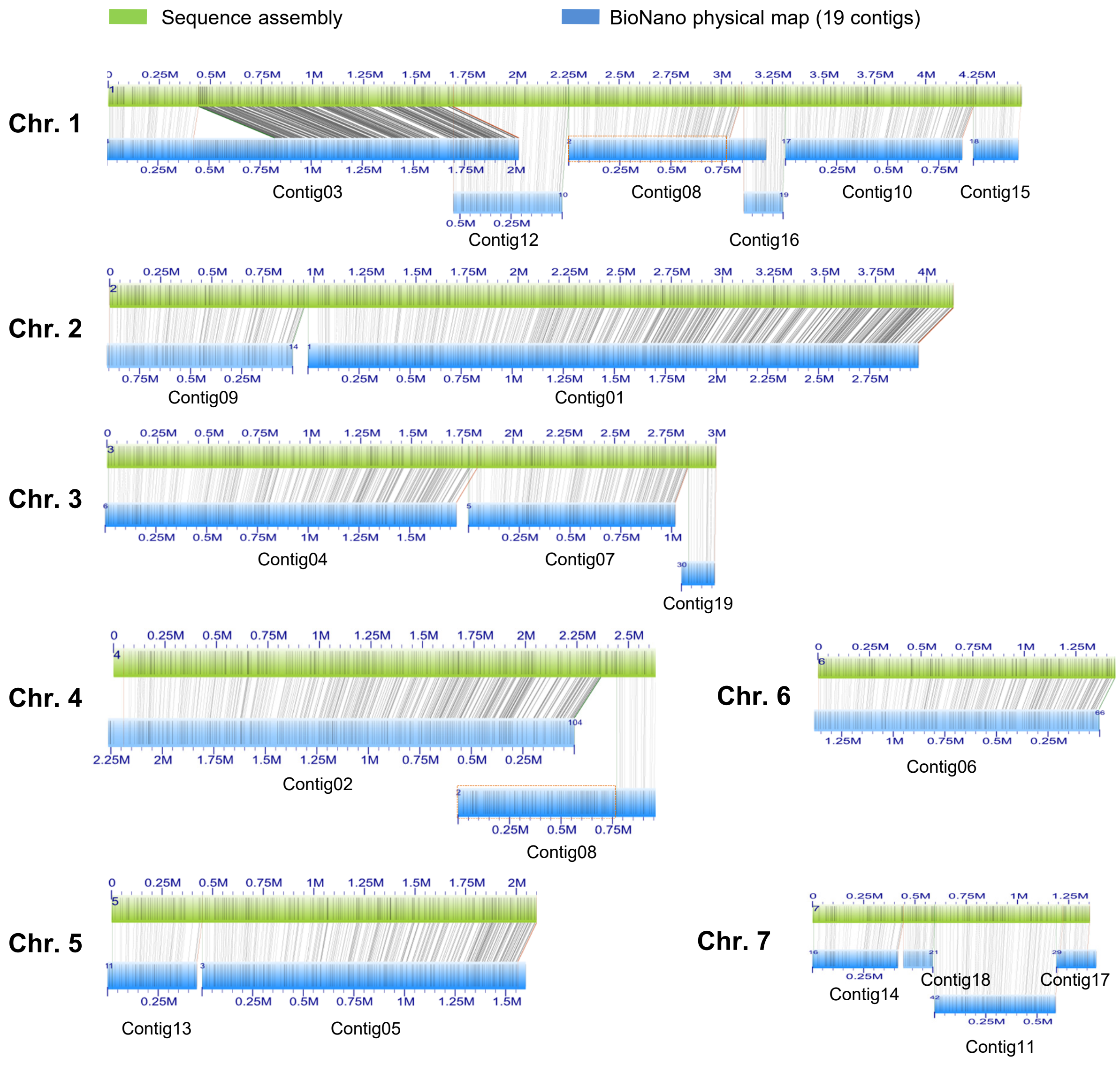

Figure S4. Sequence assembly data anchored to the BioNano physical map of *S. fibuligera* KPH12. A total of 19 Irys contigs (blue bars) were assembled based on overlapping patterns of nick locations and then anchored to each of the *in silico*-assembled seven chromosomes (green bars) of KPH12. The ~0.5-Mb gap region observed on *in silico*-assembled chromosome 1 corresponds to the rDNA repeat locus. Irys contig 8 in the red box was an artificial hybrid construct containing an internal portion of chromosome 1 and an end portion of chromosome 4, which was confirmed by PCR analysis. Black lines between the green and blue bars indicate the position of an Nt.BspQI recognition sequence on each chromosome.
