# Supplementary material for: Whole-genome de novo sequencing, combined with RNA-Seq analysis, reveals unique genome and physiological features of the amylolytic yeast Saccharomycopsis fibuligera and its interspecies hybrid
Source: Biotechnol Biofuels. 2016 Nov 11;9:246. doi: 10.1186/s13068-016-0653-4 (PMC5106798; doi:10.1186/s13068-016-0653-4)
Supplement: Supplementary file 7 — Additional file 7: Figure S6. The putative centromere regions on the S. fibuligera KPH12 and KJJ81 genomes. [file 13068_2016_653_MOESM7_ESM.pdf]

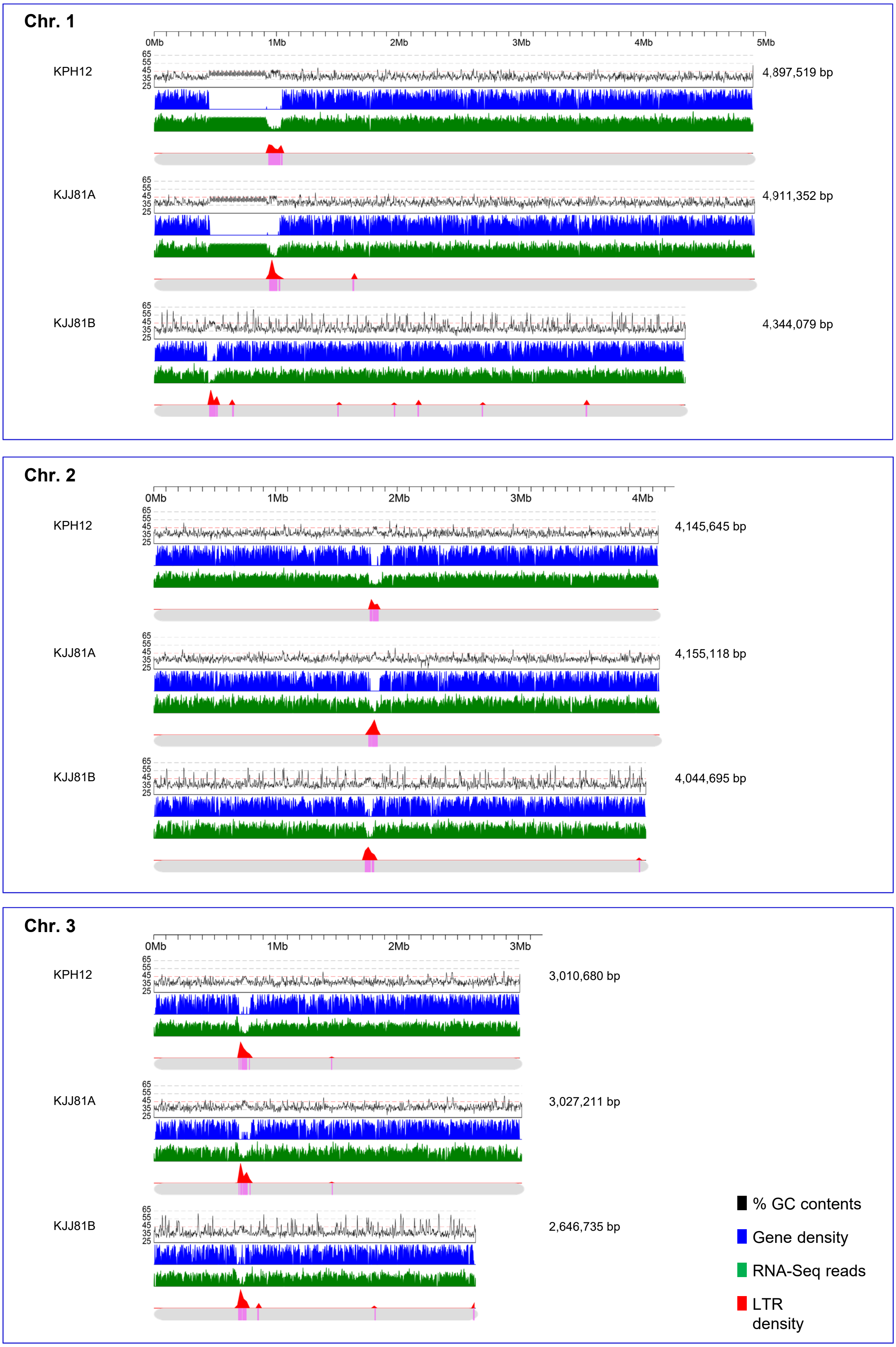

Figure S6, continued

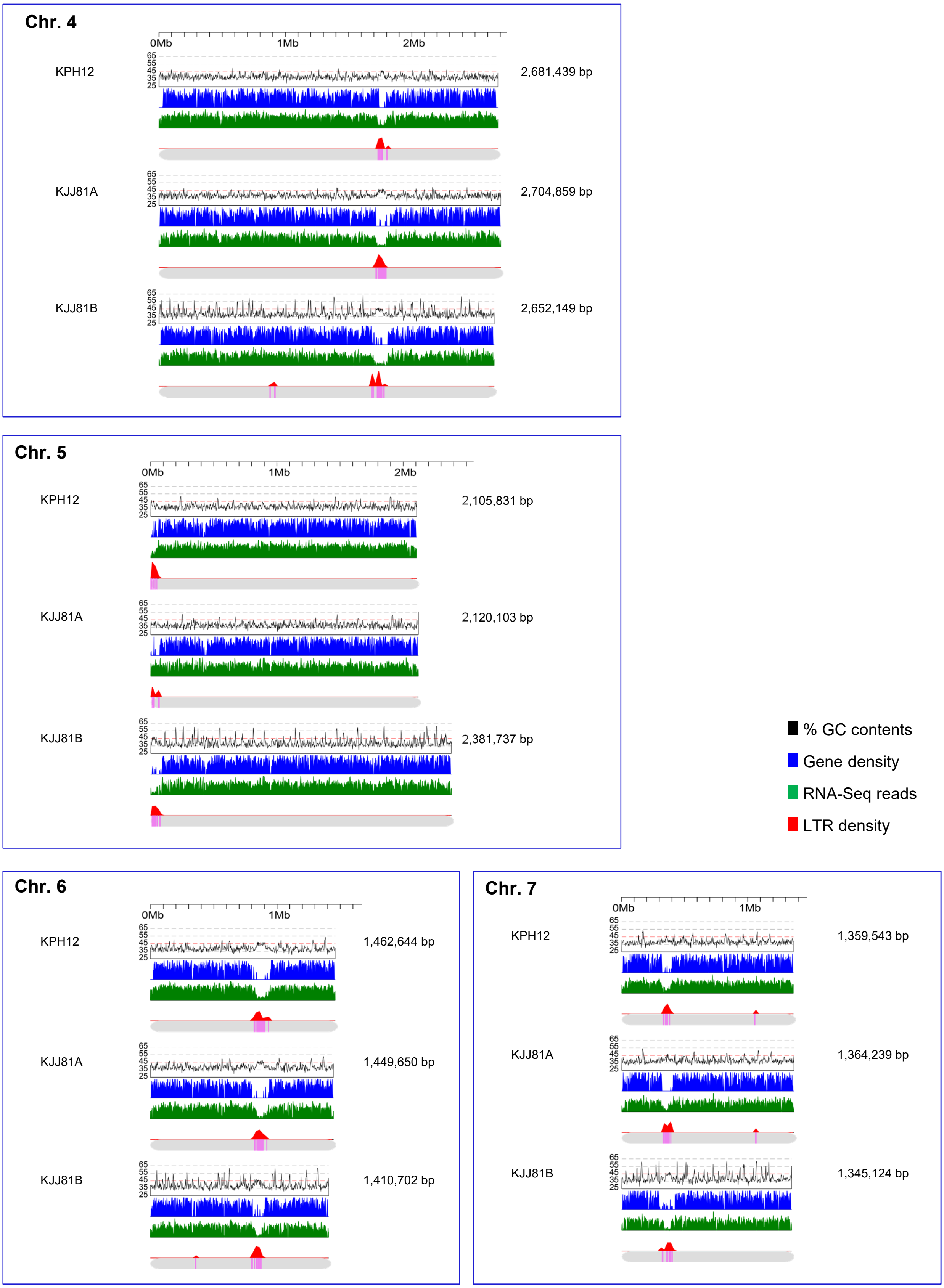

Figure S6. The putative centromere regions on the *S. fibuligera* KPH12 and KJJ81 genomes. The locations and densities of LTR retrotransposons (green) were analyzed using LTR_FINDER [1] and were plotted for each 25-kb window of the chromosomal coordinates. GC contents (black) and gene density (blue) were analyzed using the codonW program and plotted for each 2.5-kb window of the chromosomal coordinates. The log scale plots of RNA-Seq reads (green) show the transcriptome density for each 2.5-kb window of the chromosomal coordinates. TopHat2 [2] was used to map RNA-Seq reads to the PacBio genome assembly.

Reference

1. Xu Z, Wang H. LTR_FINDER: an efficient tool for the prediction of full-length LTR retrotransposons. Nucleic Acids Res. 2007; 35(Web Server issue):W265-268.
2. Trapnell C, Pachter L, Salzberg SL. TopHat: discovering splice junctions with RNA-Seq. Bioinformatics. 2009; 25:1105-1111.
