# Supplementary material for: Whole-genome de novo sequencing, combined with RNA-Seq analysis, reveals unique genome and physiological features of the amylolytic yeast Saccharomycopsis fibuligera and its interspecies hybrid
Source: Biotechnol Biofuels. 2016 Nov 11;9:246. doi: 10.1186/s13068-016-0653-4 (PMC5106798; doi:10.1186/s13068-016-0653-4)
Supplement: Supplementary file 9 — Additional file 9: Figure S8. Comparative analysis of mating type locus (MAT) organization and mating-type hormone genes between S. fibuligera subgenomes A and B. [file 13068_2016_653_MOESM9_ESM.pdf]

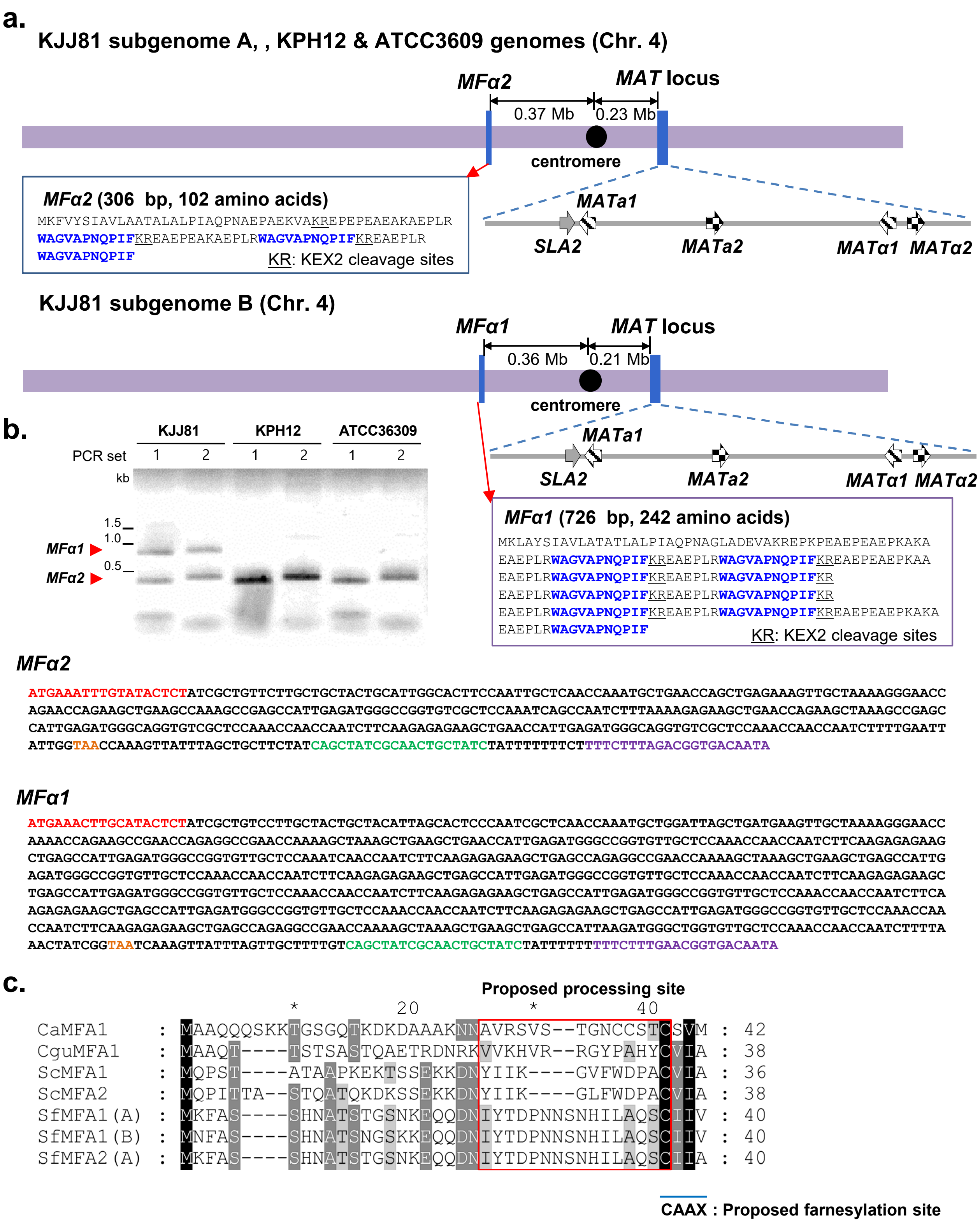

Figure S8. Comparative analysis of mating type locus (*MAT*) organization and mating-type hormone genes between *S. fibuligera* subgenomes A and B. **(a)** The *MAT* locus and *MFα* genes with their expected amino sequences. **(b)** PCR analysis to detect the presence of *MFα1* and *MFα2* genes in *S. fibuligera* isolates. PCR set 1 was performed using the primers αF1 (red, ATGAAAYTTGYATACTCT) and αR1 (green, GATAGCAGTTGCGATAGCTG), whereas PCR set 2 was performed using the primers αF1 and αR2 (purple, GTTGAATCTRTATTGTCACCG). Orange: stop codon. **(c)** Alignment of putative amino acid sequences of mating factor a (MFA) from *C. albicans* (Ca), *C. guilliermondii* (Cgu), *S. cerevisiae* (Sc), and *S. fibuligera* (Sf). C: cysteine, A: aliphatic, and X: any amino acid.
