# Supplementary material for: Whole-genome de novo sequencing, combined with RNA-Seq analysis, reveals unique genome and physiological features of the amylolytic yeast Saccharomycopsis fibuligera and its interspecies hybrid
Source: Biotechnol Biofuels. 2016 Nov 11;9:246. doi: 10.1186/s13068-016-0653-4 (PMC5106798; doi:10.1186/s13068-016-0653-4)
Supplement: Supplementary file 12 — Additional file 12: Figure S9. Comparative analysis of growth, glucose utilization, and ethanol and glycerol production between two S. fibuligera isolates and S. cerevisiae in the presence of different concentrations of glucose. [file 13068_2016_653_MOESM12_ESM.pdf]

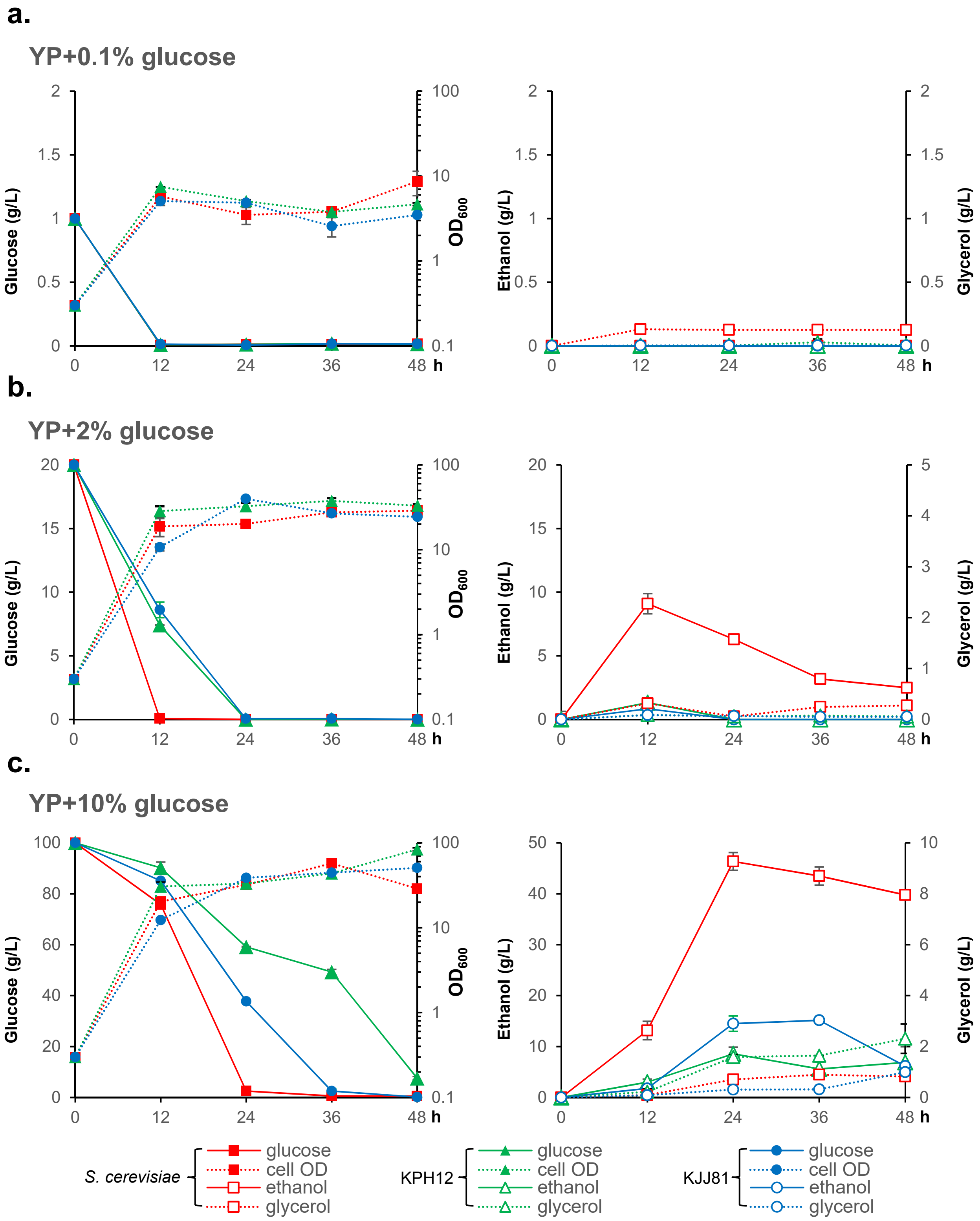

Figure S9. Comparative analysis of growth, glucose utilization, and ethanol and glycerol production between two *S. fibuligera* isolates (KPH12 and KJJ81) and *S. cerevisiae* (CEN.PK2-1C) in the presence of different concentrations of glucose. **(a)** YP+0.1% glucose **(b)** YP+2% glucose **(c)** YP+10% glucose. Shake-flask cultivation of yeast cells was carried out at 30°C for *S. cerevisiae* and 37°C for *S. fibuligera,* respectively. Concentrations of glucose, ethanol and glycerol were measured by high-pressure liquid chromatography (Thermo Fisher Scientific). Separations were achieved on Aminex HPX-87H 300-by-7.8-mm column (Bio-Rad), and peaks were detected with Refractive Index detector (Thermo Fisher Scientific). Diluted sulfuric acid (2.5 mM in water) and 0.9% acetonitrile were used as solvent at 60°C and at a 0.6 ml/min flow rate.
