# Supplementary figures and images for: Whole-genome de novo sequencing, combined with RNA-Seq analysis, reveals unique genome and physiological features of the amylolytic yeast Saccharomycopsis fibuligera and its interspecies hybrid
Source: Biotechnol Biofuels. 2016 Nov 11;9:246. doi: 10.1186/s13068-016-0653-4 (PMC5106798; doi:10.1186/s13068-016-0653-4)

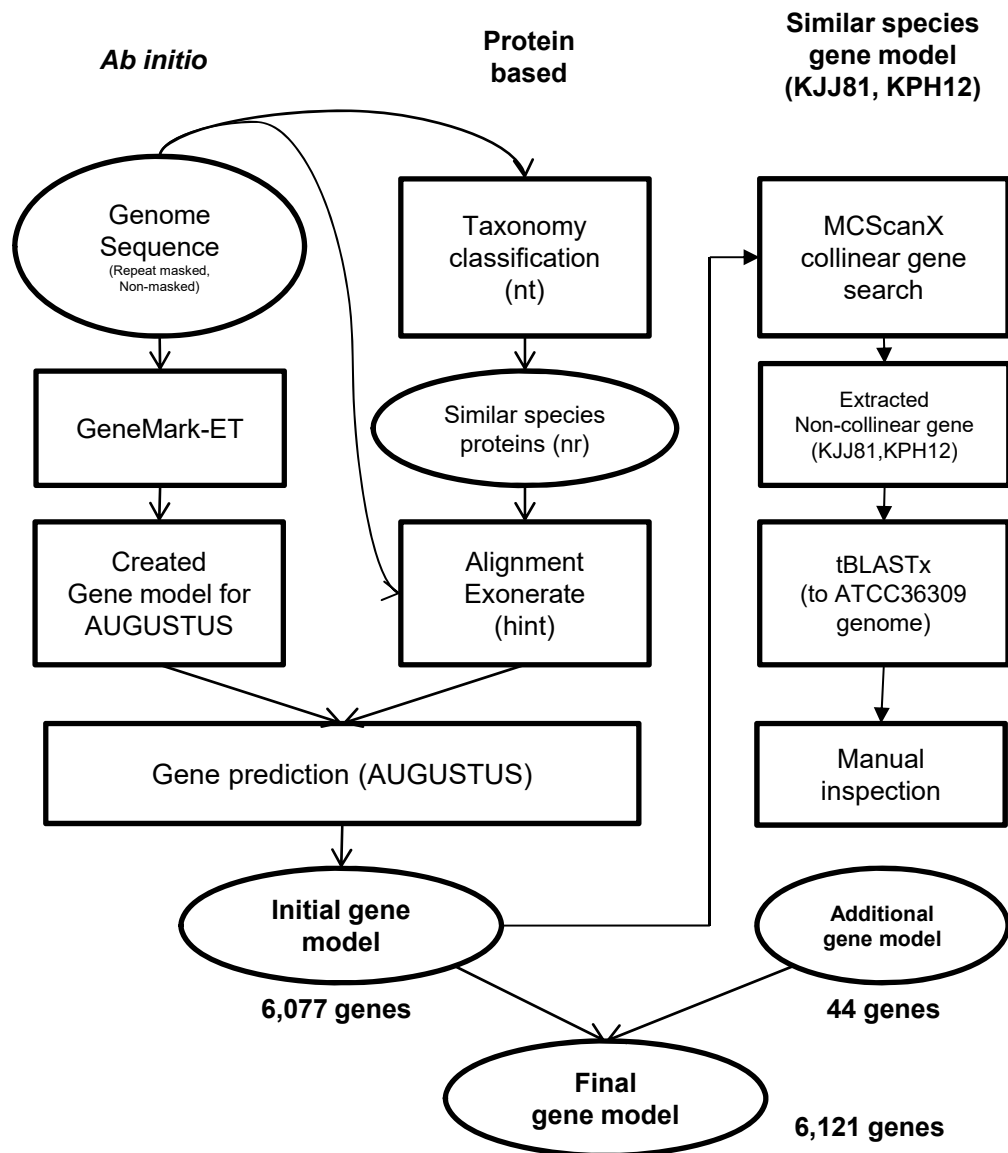

**Figure S10.** Summary of the annotation process for the *S. fibuligera* ATCC 36309 genome.

Supplement: Supplementary file 14 — Additional file 14: Figure S10. Summary of the annotation process for the S. fibuligera ATCC 36309 genome. [file 13068_2016_653_MOESM14_ESM.pdf]
