# Supplementary material for: Whole-genome de novo sequencing, combined with RNA-Seq analysis, reveals unique genome and physiological features of the amylolytic yeast Saccharomycopsis fibuligera and its interspecies hybrid
Source: Biotechnol Biofuels. 2016 Nov 11;9:246. doi: 10.1186/s13068-016-0653-4 (PMC5106798; doi:10.1186/s13068-016-0653-4)
Supplement: Supplementary file 15 — Additional file 15: Figure S11. Identification of putative centromeres and telomere regions of S. fibuligera ATCC 36309 chromosomes. [file 13068_2016_653_MOESM15_ESM.pdf]

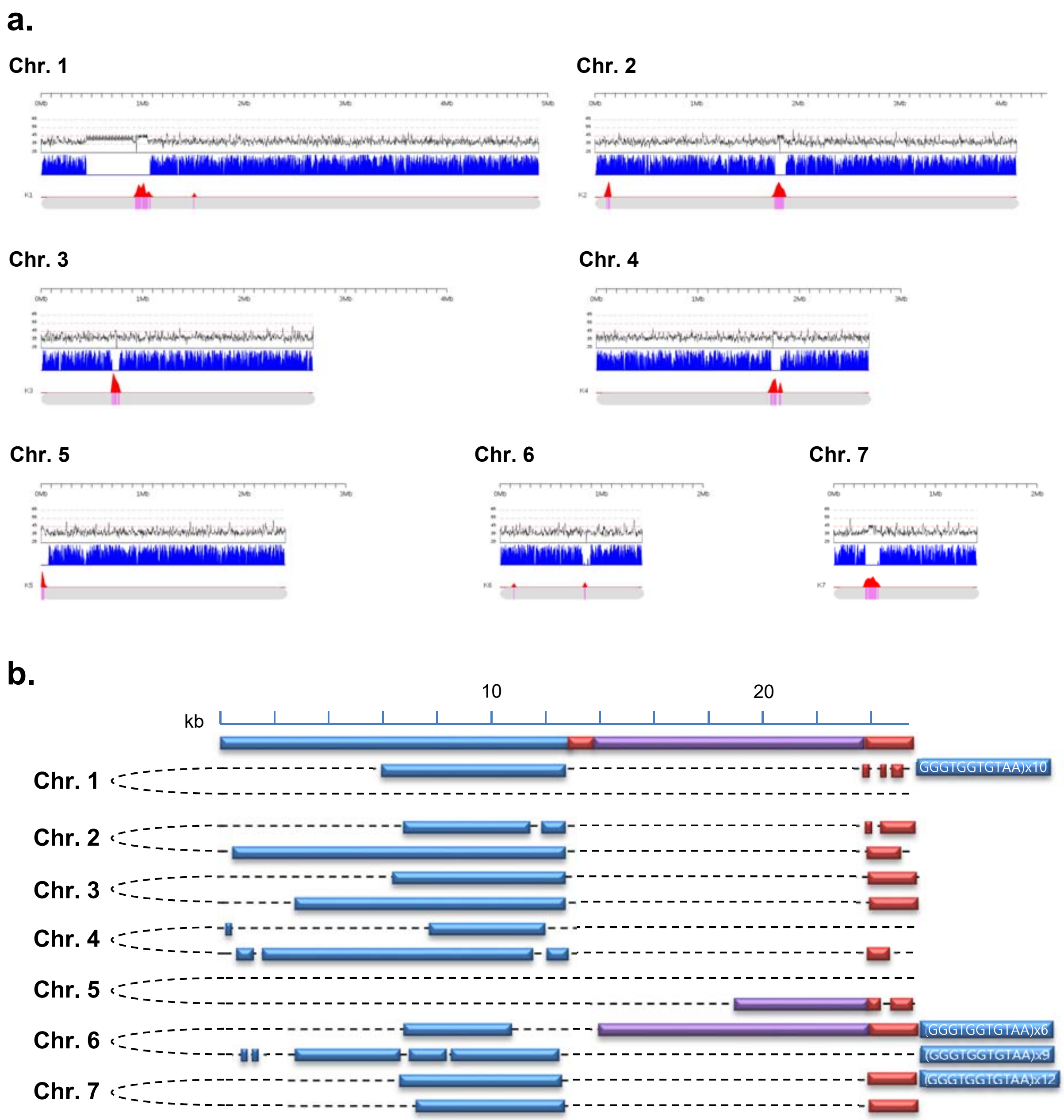

Figure S11. Identification of putative centromeres **(a)** and telomere regions **(b)** of *S. fibuliger*a ATCC 36309 chromosomes.
