# Supplementary material for: Whole-genome de novo sequencing, combined with RNA-Seq analysis, reveals unique genome and physiological features of the amylolytic yeast Saccharomycopsis fibuligera and its interspecies hybrid
Source: Biotechnol Biofuels. 2016 Nov 11;9:246. doi: 10.1186/s13068-016-0653-4 (PMC5106798; doi:10.1186/s13068-016-0653-4)
Supplement: Supplementary file 16 — Additional file 16: Figure S12. Deletion of a ~20 kb fragment containing five genes encoding SAP6, abfC, HGT1, RTA1 and YRF1-2 in the subtelomeric region of chromosome 3 of S. fibuligera ATCC 36309. [file 13068_2016_653_MOESM16_ESM.pdf]

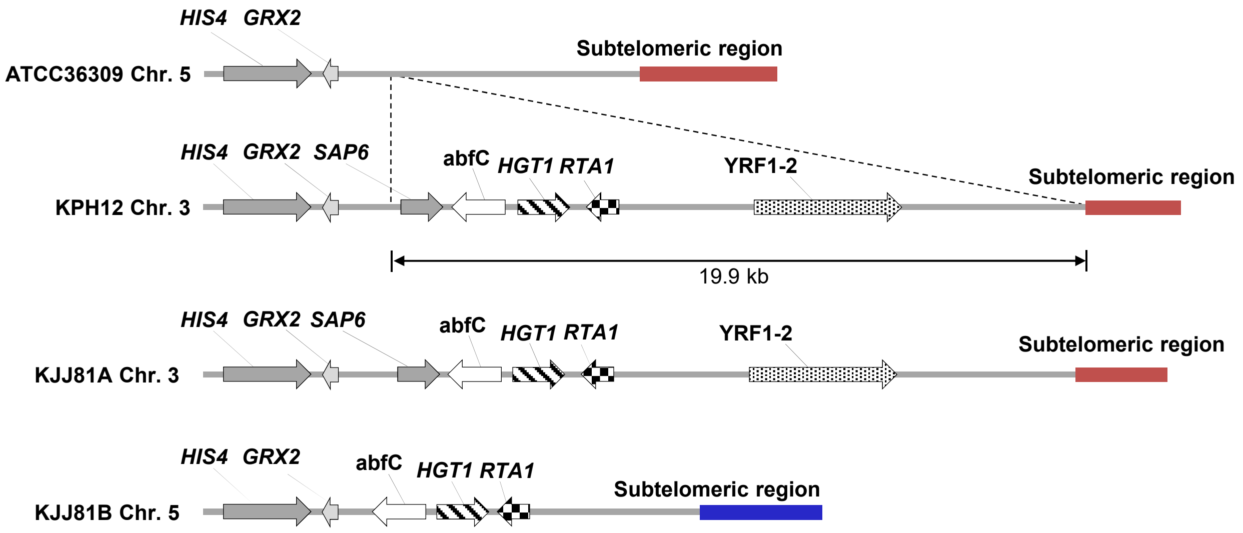

Figure S12. Deletion of a ~20-kb fragment containing five genes encoding *SAP6*, abfC, *HGT1*, *RTA1* and YRF1-2 in the subtelomeric region of chromosome 3 of *S. fibuligera* ATCC 36309.
