# Supplementary material for: Whole-genome de novo sequencing, combined with RNA-Seq analysis, reveals unique genome and physiological features of the amylolytic yeast Saccharomycopsis fibuligera and its interspecies hybrid
Source: Biotechnol Biofuels. 2016 Nov 11;9:246. doi: 10.1186/s13068-016-0653-4 (PMC5106798; doi:10.1186/s13068-016-0653-4)
Supplement: Supplementary file 17 — Additional file 17: Figure S13. Sequence divergence between subgenomes in hybrid yeast species. [file 13068_2016_653_MOESM17_ESM.pdf]

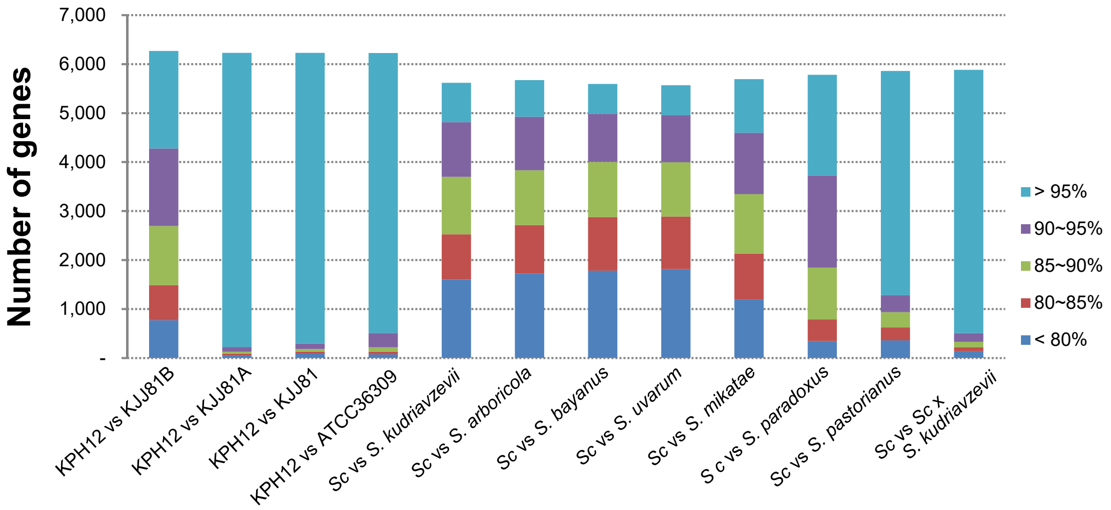

Figure S13. Sequence divergence between subgenomes in hybrid yeast species. Amino acid sequences of the genes of *S. fibuligera* KPH12 and *S. cerevisiae* were used for TBLASTN analysis of each genome sequence of the indicated yeast species.
