# Supplementary material for: Whole-genome de novo sequencing, combined with RNA-Seq analysis, reveals unique genome and physiological features of the amylolytic yeast Saccharomycopsis fibuligera and its interspecies hybrid
Source: Biotechnol Biofuels. 2016 Nov 11;9:246. doi: 10.1186/s13068-016-0653-4 (PMC5106798; doi:10.1186/s13068-016-0653-4)
Supplement: Supplementary file 18 — Additional file 18: Figure S14. Comparative growth analysis of S. fibuligera isolates at different temperatures and on different sulfur sources. [file 13068_2016_653_MOESM18_ESM.pdf]

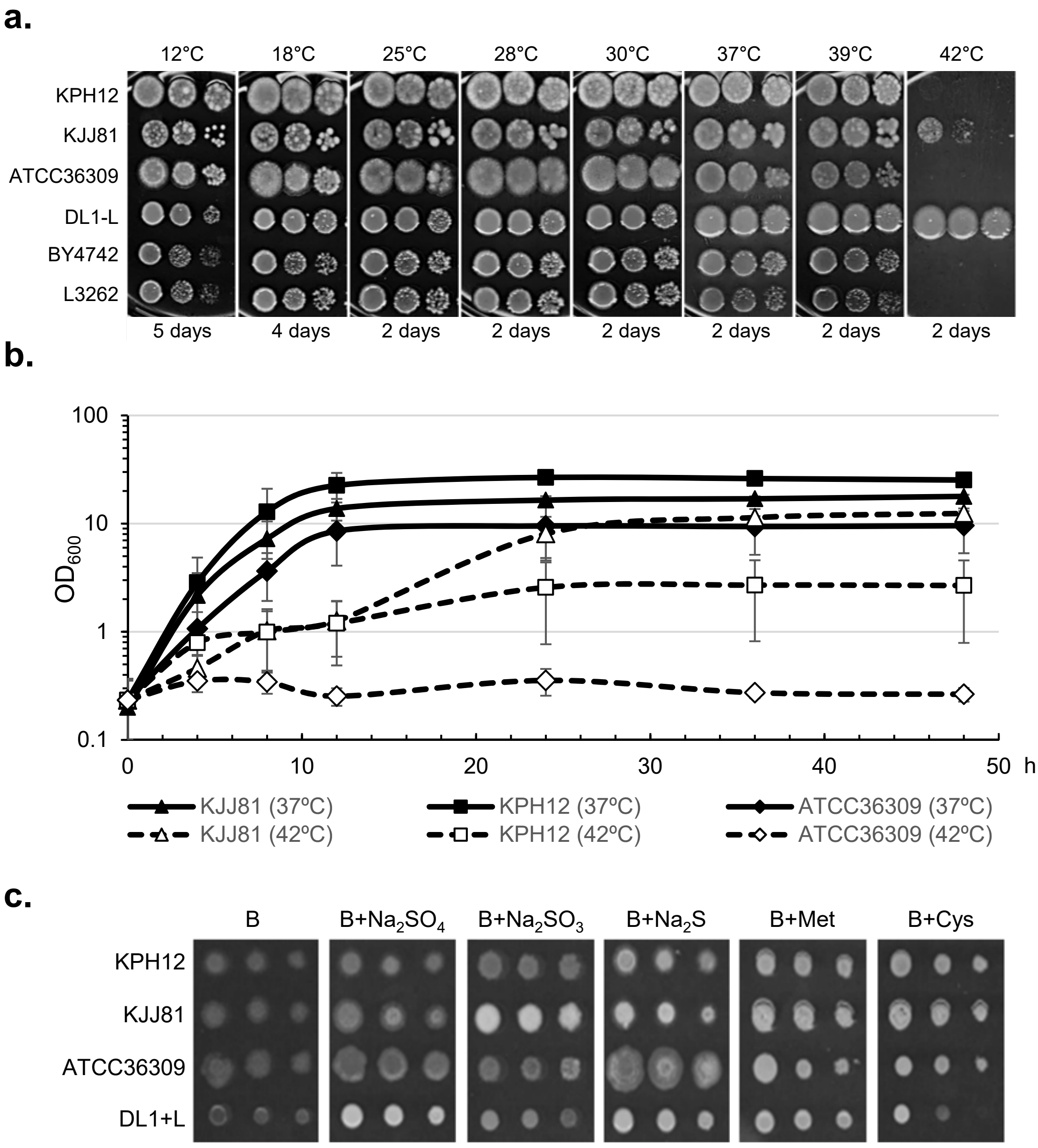

Figure S14. Comparative growth analysis of *S. fibuligera* isolates at different temperatures and on different sulfur sources. **(a)** Spotting analysis of growth at different temperatures. *S. fibuligera* isolates were serially diluted and spotted on YPD plates and cultivated at the indicated temperatures for the indicated number of days. The thermotolerant methylotrophic yeast *H. polymorpha* DL1-L and the two different *S. cerevisiae* strains BY4742 and L3262 were included for comparison. **(b)** Comparison of growth curves of *S. fibuligera* isolates cultivated in YPD liquid medium at the indicated temperatures for 49 hrs. **(c)** Spotting analysis of growth on different sulfur sources. *S. fibuligera* isolates were serially diluted and spotted on B medium plates with or without different sulfur sources, such as 1 mM Na_2SO_4, 1 mM Na_2SO_3, 1 mM Na_2S, 0.1 mM methionine (Met), or 0.1 mM cysteine (Cys).
