# Supplementary material for: Whole-genome de novo sequencing, combined with RNA-Seq analysis, reveals unique genome and physiological features of the amylolytic yeast Saccharomycopsis fibuligera and its interspecies hybrid
Source: Biotechnol Biofuels. 2016 Nov 11;9:246. doi: 10.1186/s13068-016-0653-4 (PMC5106798; doi:10.1186/s13068-016-0653-4)
Supplement: Supplementary file 6 — Additional file 6: Figure S5. Mitochondrial genomes of S. fibuligera KPH12 and KJJ81. [file 13068_2016_653_MOESM6_ESM.pdf]

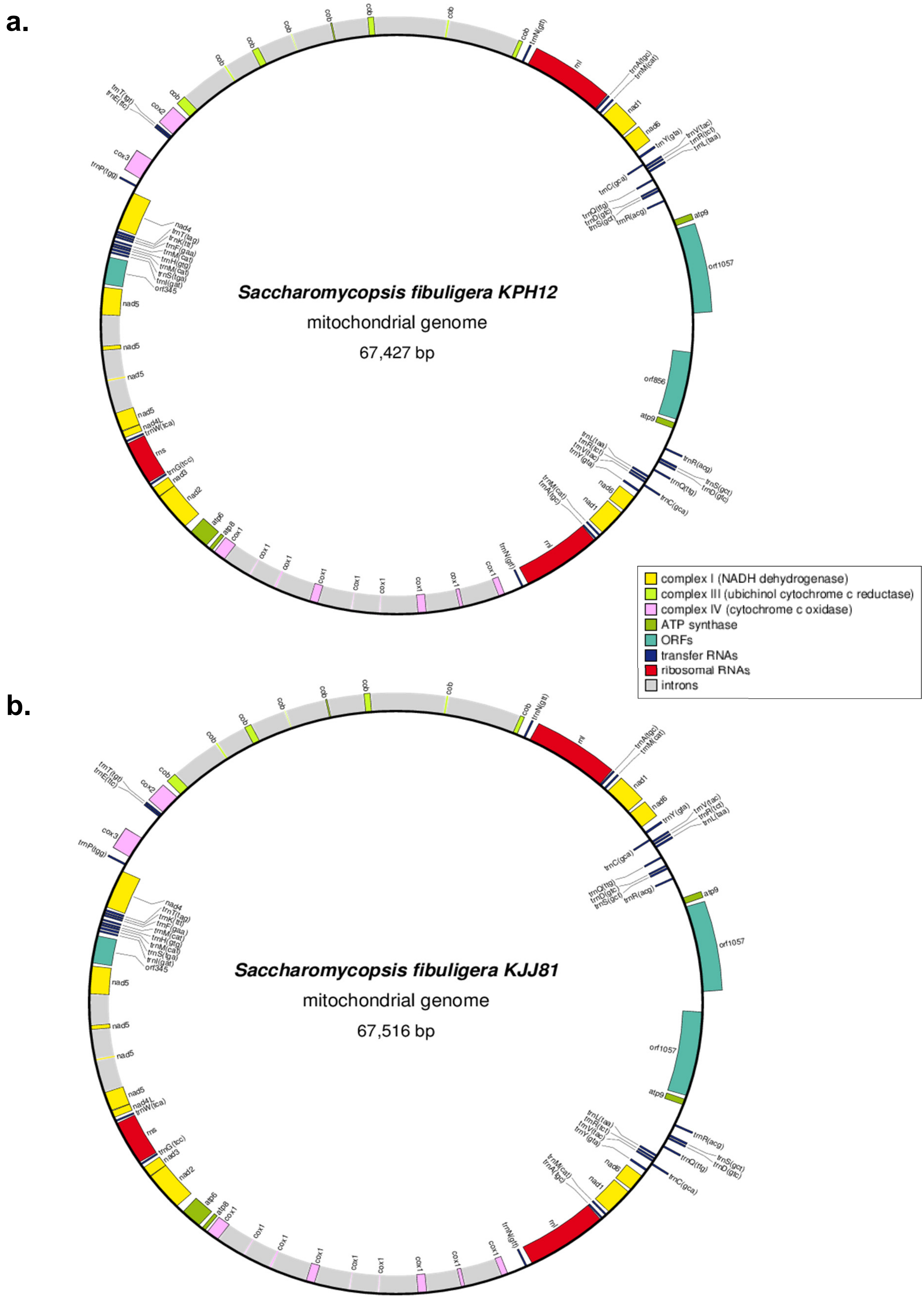

Figure S5. Mitochondrial genomes of *S. fibuligera* KPH12 **(a)** and KJJ81 **(b)**. The assembled sequences were analyzed using the MFannot program. The annotated genes are listed in Additional file 1: Table S6.
