# Supplementary material for: Whole-genome de novo sequencing, combined with RNA-Seq analysis, reveals unique genome and physiological features of the amylolytic yeast Saccharomycopsis fibuligera and its interspecies hybrid
Source: Biotechnol Biofuels. 2016 Nov 11;9:246. doi: 10.1186/s13068-016-0653-4 (PMC5106798; doi:10.1186/s13068-016-0653-4)
Supplement: Supplementary file 8 — Additional file 8: Figure S7. Identification of repeat sequences at both ends of the S. fibuligera KPH12 and KJJ81 chromosomes. [file 13068_2016_653_MOESM8_ESM.pdf]

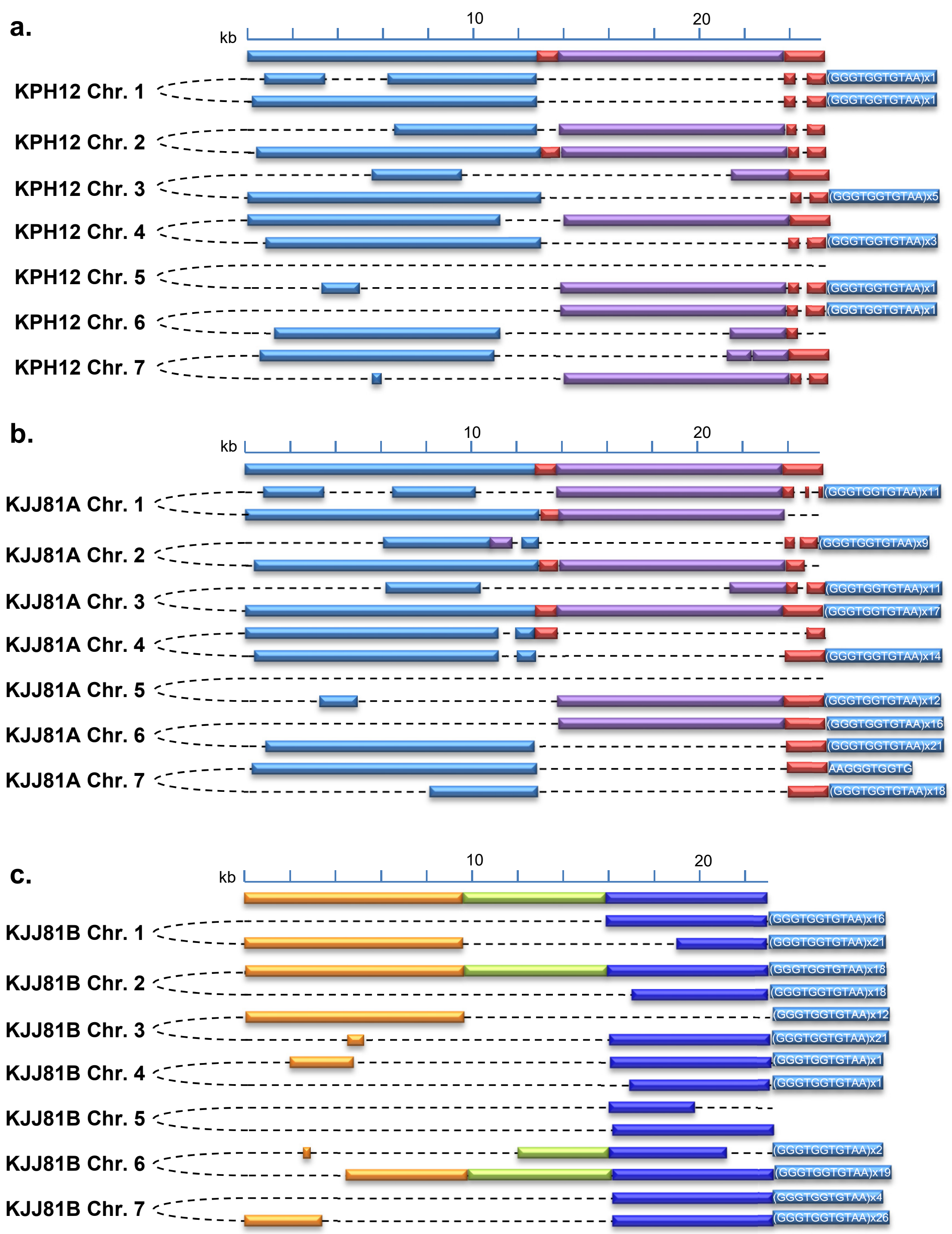

Figure S7. Identification of repeat sequences at both ends of the *S. fibuligera* KPH12 and KJJ81 chromosomes. Each colored box represents blocks of subtelomeric regions with different repeat sequences. The putative telomeric sequence "GGGTGGTGTAA" of *S. fibuligera* is shown at the end of each chromosome. **(a)** The largest repeat block of subtelomeric regions found in chromosome 3 of KJJ81 subgenome A consisted of a front block of ~14 kb and an end block of ~12 kb. Interestingly, the ~1.0-kb end of the front block was observed to be identical to a portion of the ~1.7-kb end block. The subtelomeric regions of other chromosomes in KJJ81 subgenome A also possessed the same repeat blocks but generally retained partial structure. **(b)** The chromosomes of KPH12 displayed subtelomeric organization quite similar to that of KJJ81 subgenome A. **(c)** The largest repeat blocks of subtelomeric regions in KJJ81 subgenome B, which were identified in chromosome 2, consisted of the 9.6-kb front block, the 6.1-kb middle block, and the 7.1-kb end block.
